# High‐Load Core@Shell Nanocarriers with Irinotecan and 5‐Fluorouracil for Combination Chemotherapy in Colorectal Cancer

**DOI:** 10.1002/smsc.202400196

**Published:** 2024-08-19

**Authors:** Silke Notter, Dolma Choezom, Titus Griebel, Fernanda Ramos‐Gomes, Wiebke Möbius, Tiago De Oliveira, Lena‐Christin Conradi, Frauke Alves, Claus Feldmann

**Affiliations:** ^1^ Institute of Inorganic Chemistry Karlsruhe Institute of Technology (KIT) Engesserstraße 15 76131 Karlsruhe Germany; ^2^ Clinic for Haematology and Medical Oncology University Medical Center Goettingen (UMG) Robert Koch Straße 40 37075 Goettingen Germany; ^3^ Max‐Planck‐Institute for Multidisciplinary Sciences (MPI‐NAT), Department of Neurogenetics City Campus, Hermann‐Rein‐Straße 3 37075 Goettingen Germany; ^4^ Department of General, Visceral and Pediatric Surgery University Medical Center Goettingen (UMG) Robert‐Koch‐Straβe 40 37075 Göttingen Germany; ^5^ Institute for Diagnostic and Interventional Radiology University Medical Center Goettingen (UMG) Robert Koch Straße 40 37075 Goettingen Germany

**Keywords:** 5‐fluorouracil, colorectal cancer, drug delivery, irinotecan, tumor combination therapy

## Abstract

Colorectal cancer (CRC) is the third most common cancer type and second leading cause of cancer‐related deaths worldwide, requiring novel drug‐delivery concepts. ITC@ZrO(TocP)/ZrO(FdUMP) core@shell nanocarriers (designated ITC‐FdUMP‐NC) with the clinically relevant chemotherapeutics irinotecan (ITC) and fluoro‐2′‐deoxyuridine‐5′‐phosphate (FdUMP) (active derivative of 5′‐fluorouracil/5‐FU) are a new type of nanocarrier with high drug payload (22 wt% of lipophilic ITC: particle core; 10 wt% of hydrophilic FdUMP: particle shell). The nanocarriers are tested in different CRC cell lines, a normal cell line, and rectal cancer patient‐derived organoids (PDOs). Fluorescence‐labeled nanocarriers show efficient uptake by all CRC cells and allow to distinctly track the intracellular trafficking toward endolysosomal compartments. Although free chemotherapeutic drugs exhibit a greater potency in 2D cell cultures, ITC‐FdUMP‐NC demonstrate equivalent cytotoxic efficacies as the freely dissolved drugs in the more complex 3D rectal cancer PDOs. The sustained drug‐release profile of the nanocarriers contrasts favorably with conventional free drugs, potentially enhancing the therapeutic outcome in vivo. With a chemotherapeutic cocktail comparable to the clinically applied FOLFIRI (ITC + 5‐FU), the ITC‐FdUMP‐NC represent a novel type of nanocarrier with high anti‐tumor effect and high drug payload, offering a promising strategy to circumvent chemoresistance and to improve therapy efficacy in vivo with less side effects.

## Graphical abstract

A clinically relevant drug cocktail of irinotecan (ITC) and 5‐fluorouridine (5‐FU) is combined in a single nanocarrier with high drug payload (32%). The nanocarriers show efficient uptake and high efficacy in different human colon cancer and human rectal cancer cell lines as well as rectal cancer patient‐derived organoids, also allowing to elucidate the membrane‐trafficking pathway.

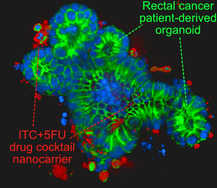

## 1 Introduction

Colorectal cancer (CRC) is the third most common cancer type worldwide (after lung and prostate cancer) with more than 1.9 million new cases in 2020, causing about 900 000 cancer‐related deaths per year.^[^ ^1^ ^]^ The overall incidence of CRCs (i.e., adenocarcinoma of colon and rectum) has decreased among older adults as a consequence of early detection through screening and primary prevention strategies such as optimizing a healthy lifestyle, and avoiding risk factors, however, at the same time, incidence is increasing among younger adults.^[^ ^2^ ^]^ Adenocarcinomas of the colon and rectum make up the majority of all CRC cases.^[^ ^3^ ^]^ Although CRC has a favorable prognosis when diagnosed in early stages, it is still the second leading cause of cancer‐related deaths worldwide since patients are often diagnosed at an advanced stage resulting in a grim prognosis. More than 20% of the patients present with metastatic CRC and around 50% of patients with localized disease will develop metastases, mainly in the liver and the peritoneum.^[^ ^4^ ^]^ Only about 25% of these patients are amenable to liver‐directed therapies such as resection, ablation, or embolization and approximately one third experience disease relapse following curative‐intent surgical resection and chemotherapy.^[^ ^5^ ^]^

In the case of non‐resectable metastatic CRC, systemic chemotherapy is the first‐line treatment option. A combination therapy of folinic acid‐fluorouracil‐oxaliplatin (so‐called FOLFOX), folinic acid‐fluorouracil‐oxaliplatin‐irinotecan (so‐called FOLFOXIRI), or folinic acid‐5‐fluorouracil‐irinotecan (so‐called FOLFIRI) is most often applied.^[^ ^6^ ^]^ Chemotherapy, in combination with irradiation, is also administered to shrink locally advanced rectal adenocarcinomas before surgery. High inter‐patient variability and high spatial heterogeneity are further features of CRC.^[^ ^7^ ^]^ This is addressed by using a combination of chemotherapeutic drugs in varying dosages and concentrations, by which different cellular processes required for the rapid proliferation of cancer cells are disrupted. Moreover, the personalized needs for a specific tumor subtype and patient can be adjusted.^[^ ^8^ ^]^ The benefit of this currently intravenously applied combination chemotherapy is often limited by poor aqueous solubility, suboptimal pharmacokinetics, low drug accumulation at the tumor site, high off‐target localization being harmful for healthy tissue causing severe side effects.

To address the aforementioned limitations, we intend to use nanocarriers that selectively accumulate at the site of the tumor due to the enhanced permeability and retention (EPR) effect for drug release, resulting either in a higher efficacy and/or lower side effects as compared to the clinically applied dissolved drugs.^[^ ^8^ ^]^ Since combination regimens often suffer from varying pharmacokinetics among different chemotherapeutic drugs, nanocarriers can offer the possibility to deliver various drugs simultaneously and unify the pharmacokinetics of each drug. Furthermore, nanocarriers can protect the drug from rapid systemic degradation and metabolism under physiological conditions. Especially, this is relevant for prodrugs such as the topoisomerase I inhibitor irinotecan (ITC), that becomes rapidly activated in vivo to its extremely active metabolite SN‐38,^[^ ^9^ ^]^ and for the widely used anti‐cancer drugs 5‐fluorouracil (5‐FU) and 5‐fluorodeoxyuridine that form in vivo the active form fluorodeoxyuridylate, also known as 5‐fluoro‐2′‐deoxyuridine 5′‐monophosphate (FdUMP), that acts as a suicide inhibitor of thymidylate synthase (TS), thereby inhibiting the deoxynucleotide biosynthesis.^[^ ^10^ ^]^

Although chemotherapeutic nanocarriers such as non‐PEGylated liposomal doxorubicin (Myocet) or PEGylated liposomal doxorubicin (CAELYX, DOXIL) already reached clinical approval,^[^ ^11^ ^]^ current nanocarrier concepts often suffer from low drug loads (per total mass of the nanocarrier) and are usually limited to a single drug,^[^ ^12^ ^]^ whereas two drugs in a single nanocarrier with >20 wt% of total nanocarrier mass are rare until now.^[^ ^13^ ^]^ Aiming at nanocarriers with high drug load, we were recently successful with gemcitabine monophosphate (GMP) containing nanocarriers (76 wt% GMP of total nanocarrier mass) to treat pancreatic cancer,^[^ ^14^ ^]^ and bedaquiline (BDQ) containing nanocarriers (99 wt% of total nanocarrier mass) to treat tuberculosis.^[^ ^15^ ^]^ A combination of drugs with different chemical properties (i.e., functional groups, solubility, hydro‐/lipophilicity, acidity, molecular weight) in a single nanocarrier, however, is much more difficult. To this concern, we here show a combination of the lipophilic chemotherapeutics ITC and the hydrophilic fluoro‐2′‐deoxyuridine‐5′‐phosphate (FdUMP) as the active derivative of 5′‐fluorouracil (5‐FU) in a single nanoparticle with loads of 22 wt% ITC and 10 wt% FdUMP of the total nanoparticle mass. This offers the chance to realize chemotherapeutic cocktails with sufficient drug concentration as compared to the clinically applied cocktails of dissolved drugs.

## 2 ITC@ZrO(TocP)/ZrO(FdUMP) Core@Shell Nanocarriers

### 2.1 Material Concept and Synthesis

**Figure 1:**
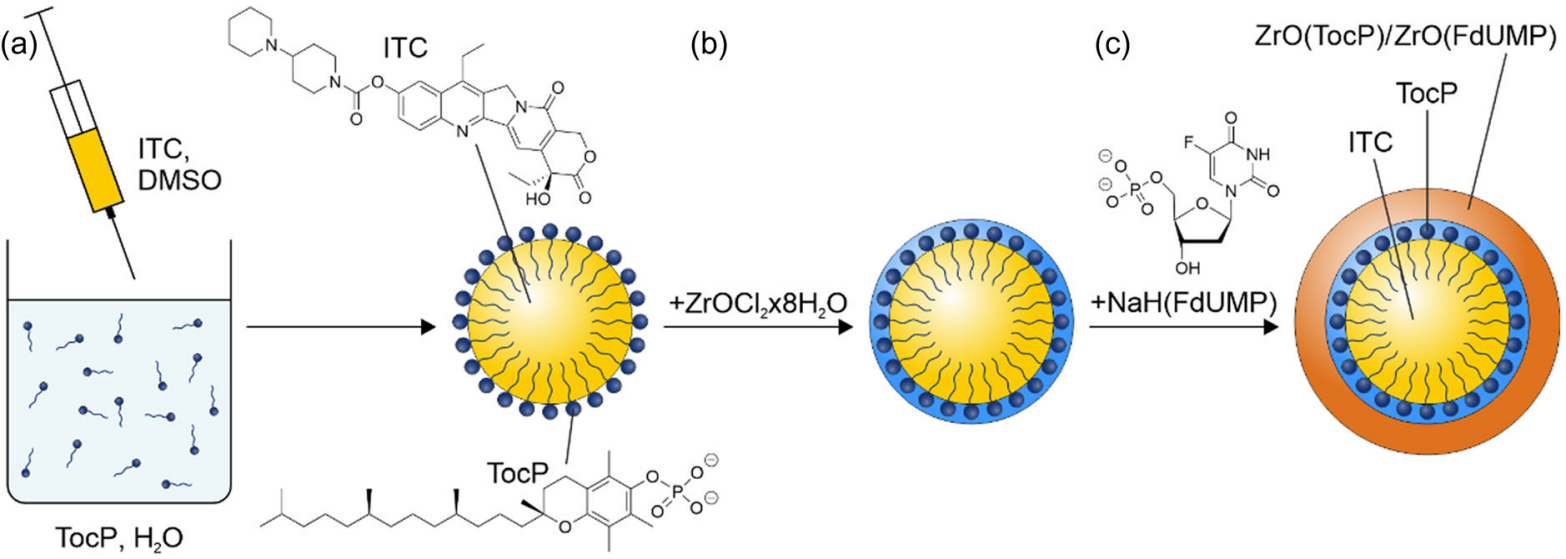
Scheme illustrating the synthesis of ITC@ZrO(TocP)/ZrO(FdUMP) core@shell nanocarriers with a) solvent‐antisolvent approach to obtain the ITC nanoparticle core, b) stabilization with [TocP]^2−^ and [ZrO]^2+^, and c) formation of the ZrO(FdUMP) shell.

**Figure 2:**
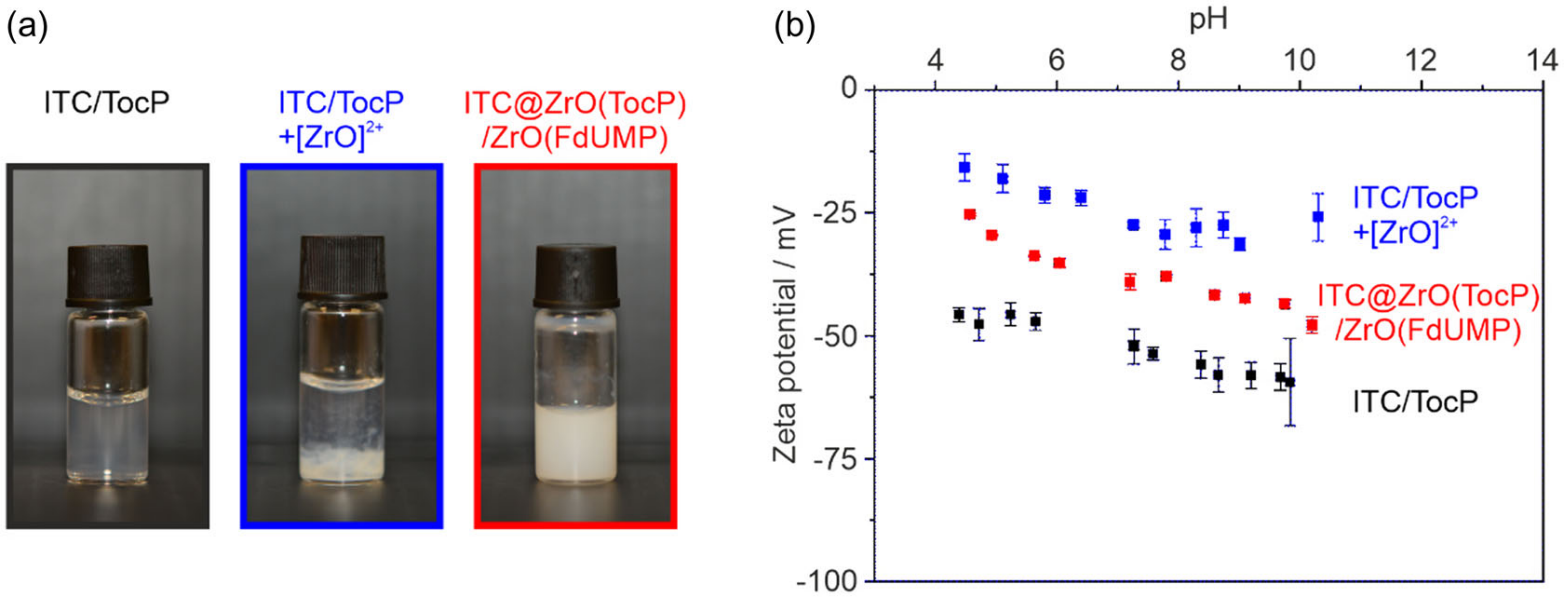
Colloidal properties of TocP‐stabilized ITC nanoparticles, TocP‐stabilized ITC nanoparticles after addition of [ZrO]^2+^, ITC@ZrO(TocP)/ZrO(FdUMP) nanocarriers: a) photos of suspensions; b) pH‐dependent zeta‐potential analysis.

The initial challenge of combining ITC and FdUMP in a single nanocarrier relates to their different chemical properties and specifically to their different solubility. Whereas ITC is lipophilic and with poor solubility in water (0.11 mg mL^−1^),^[^ ^16^ ^]^ FdUMP is hydrophilic and well‐soluble in water (>50 mg mL^−1^).^[^ ^17^ ^]^ Therefore, different strategies for synthesis are necessary to make both drugs insoluble in water and to realize dual‐drug nanocarriers (**Figure** **1**). First of all, ITC nanoparticles were prepared using a so‐called solvent‐antisolvent approach (Figure 1a). In this regard, ITC was dissolved in dimethylsulfoxide (DMSO) as the “solvent”. This solution was injected into water as the “antisolvent”. Whereas DMSO is mixable with water in any ratio, ITC is insoluble in water and precipitates immediately after injection. Although ITC nanoparticles are instantaneously obtained, they are colloidally instable and show fast agglomeration since the interaction of the lipophilic particle surface with each other is preferred over the interaction with water as highly polar solvent. Therefore, some additional measures are required. Thus, diluted ammonium acetate (65 mm) was used instead of pure water since the presence of the salt – according to the salting‐out effect^[^ ^18^ ^]^ – increases the ionic strength of water and decreases the solubility of ITC in the antisolvent. Moreover, tocopherolphosphate (TocP, a derivative of vitamin E) was added as a surfactant to moderate the contact between the lipophilic ITC surface and water as a polar solvent (Figure 1b). Finally, cooling (ice bath) and intense mixing support a rapid nucleation and formation of small ITC nanoparticles. After centrifugation/redispersion from/in water to remove excess starting materials and salts, a colloidally stable suspension of TocP‐stabilized ITC nanoparticles was obtained with negative surface charging of −59 to −46 mV at pH 4–10 (**Figure** **2**).

After the formation of the TocP‐stabilized ITC nanoparticles, an aqueous solution of ZrOCl_2_×8 H_2_O was slowly added over 30 min to the suspension. As a result, [ZrO]^2+^ cations bind to the phosphate groups of TocP (Figure 1b). The decrease of the highly negative surface charge of TocP‐stabilized ITC nanoparticles (−59 to −46 mV, pH 4–10) after addition of [ZrO]^2+^ can be followed qualitatively by the colloidal stability, which now leads to a precipitation of the nanoparticles (Figure 2a), and quantitatively based on zeta‐potential analyzes (−26 to −16 mV, pH 4–10, Figure 2b). Finally, a solution of NaH(FdUMP) was added slowly (2 min) to the suspension (Figure 1c). This results in the formation of ZrO(FdUMP) as a shell around the initial ITC nanoparticle, which now serves as the core. [ZrO]^2+^ is here used to transform the well soluble [FdUMP]^2−^ into ZrO(FdUMP), which is poorly soluble in water.^[^ ^19^ ^]^ After addition of FdUMP and formation of ZrO(FdUMP), furthermore, the resulting ITC@ZrO(TocP)/ZrO(FdUMP) nanocarrier exhibits a surface termination with FdUMP, which results in a re‐established higher negative surface charge (−48 to −25 mV, pH 4–10, Figure 2b) and visually in a good colloidal stability (Figure 2a). Here, it should also be noticed that the visual scattering intensity of the ITC@ZrO(TocP)/ZrO(FdUMP) core@shell nanocarriers already indicates a larger particle size compared to the initial ITC nanoparticles (Figure 2a). After final purification via centrifugation/redispersion to remove remaining starting materials and salts, the as‐prepared ITC@ZrO(TocP)/ZrO(FdUMP) suspensions are stable over several weeks (Figure S1 and S2, Supporting Information).

**Figure 3:**
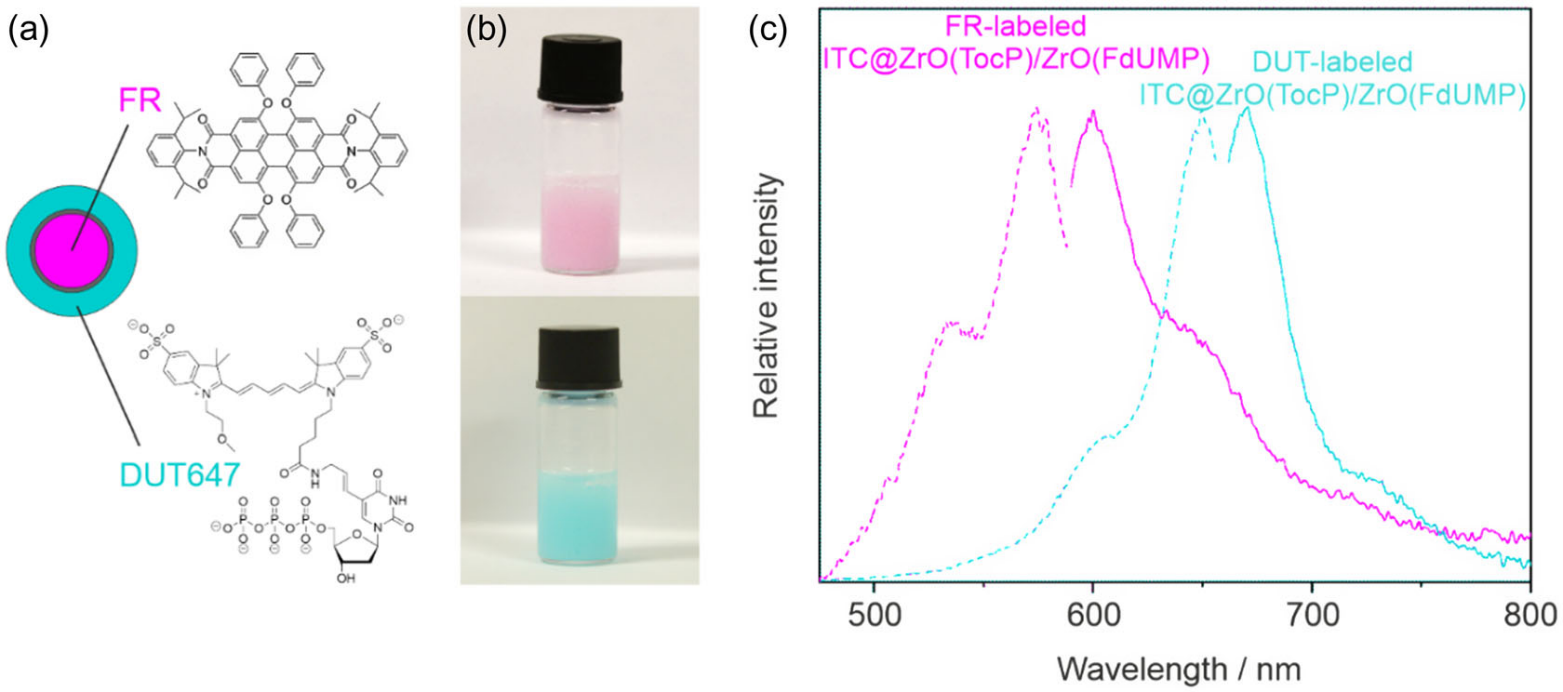
Fluorescence of FR‐labeled (1.7 mol%) and DUT647‐labeled (1.0 mol%) ITC@ZrO(TocP)/ZrO(FdUMP) nanocarriers: a) scheme of core@shell nanocarrier structure and the localization of the respective dye; b) photos of suspensions; c) excitation and emission spectra with *λ*_ex_: 575, *λ*_em_: 615 nm for FR and *λ*_ex_: 647, *λ*_em_: 670 nm for DUT647.

To enable the ITC@ZrO(TocP)/ZrO(FdUMP) nanocarriers for fluorescence tracking in vitro and/or in vivo, they can be labeled either in the lipophilic ITC core or in the hydrophilic ZrO(FdUMP) shell. A suitable fluorescent dye just needs to be added with small quantities (1–2 mol%) to the respective ITC or FdUMP solution. Specifically, we used the lipophilic perylene derivative fluorescence red (FR) together with ITC and/or the hydrophilic triphosphate‐functionalized dye DY‐647P1‐aadUTP (DUT647) together with FdUMP (**Figure** **3**a). Both fluorescent dyes show intense emission. Their incorporation in the ITC@ZrO(TocP)/ZrO(FdUMP) nanocarriers is visually indicated already by the color of the suspension. Thus, FR‐labeled nanocarriers have a red color, whereas DUT647‐labeled nanocarriers have a bluish‐green color (Figure 3b). Furthermore, the presence of the fluorescence dyes was validated by fluorescence spectroscopy. Accordingly, FR‐labeled nanocarriers show excitation at 480–580 nm (*λ*_max_: 575 nm) and emission at 580–700 nm (*λ*_max_: 615 nm); DUT647‐labeled nanocarriers show excitation at 580–660 nm (*λ*_max_: 647 nm) and emission at 660–750 nm (*λ*_max_: 670 nm) (Figure 3c).

### 2.2 Composition and Structure

The chemical composition of the as‐prepared ITC@ZrO(TocP)/ZrO(FdUMP) core@shell nanocarriers was evaluated by X‐ray powder diffraction (XRD), Fourier transform infrared (FT‐IR) spectroscopy, total organics combustion with thermogravimetry (TG), elemental analysis (EA), and photometry. Due to the high costs of NaH(FdUMP) (100 mg á 4,000 €), we have performed the analytical characterization with ITC@ZrO(TocP)/ZrO(UMP) core@shell nanocarriers since Na_2_(UMP) is much cheaper (100 mg á 5 €). In difference to FdUMP, UMP does not contain any fluorine and is not cytotoxic, which, however, does not affect the particle size or the overall composition of the nanocarriers.

XRD indicates the nanocarriers to be amorphous (Figure S3a, Supporting Information). In regard of the large volume of the drug anions and the low temperature of synthesis, such finding is expected.^[^ ^11^, ^12^, ^16^ ^]^ For drug release, however, amorphous nanocarriers are considered to be advantageous as the dissolution rate is often enhanced in comparison to crystalline drug nanocarriers.^[^ ^17^ ^]^ FT‐IR spectra show intense and P = O vibrations (1100, 990 cm^−1^), which originate from TocP and UMP, as well as C = O vibrations (1680 cm^−1^) stemming from UMP (Figure S3b, Supporting Information). Furthermore, the presence of ITC is indicated by pyridine‐related vibrations (*ν*(C = N), *ν*(C = C): 1620–1600 cm^−1^). TG shows total organic combustion up to 800 °C with a total mass loss of 68 wt% due to the decomposition of ITC, TocP, and UMP (Figure S4a, Supporting Information). The solid residue (33 wt%) was identified by XRD to be ZrO_2_ and ZrP_2_O_7_ (Figure S4b, Supporting Information). EA results in 45.4% C, 6.4% H and 3.4% N. Hereof, the nitrogen content can be assigned to ITC and UMP. Based on the amount and ratio of the starting materials (i.e., 3.92 mmol ITC, 7.03 mmol Na_2_(TocP), 3.07 mmol Na_2_(UMP)), a content of 54.0 wt% C, 6.6 wt% H, 3.6 wt% N and a total organic content of 71% can be calculated, which are in agreement with the experimental data. The experimentally observed lower C content can be ascribed to an incomplete combustion in the presence of high loads of phosphate (see Supporting Information for details). Specifically, the N content and the ratio of ITC and UMP are well in agreement with the expectation and also confirmed by photometry.

Finally, the drug load of the ITC@ZrO(TocP)/ZrO(UMP) nanocarriers was examined by photometry. To this concern, UV‐Vis spectra were recorded (Figure S5a, Supporting Information). Herein, the characteristic absorbance of ITC (360 nm) and UMP (260 nm) was used for comparison with reference samples using the Lambert–Beer law. The reference samples with ITC/TocP suspended in water and UMP dissolved in water show a good linear correlation of optical absorption and concentration (Figure S5b, Supporting Information). With the resulting calibration, photometry results in 2.9 mg mL^−1^ of ITC and 1.3 mg mL^−1^ of UMP at a particle concentration of 13 mg mL^−1^, which corresponds to drug loads of 22 wt% ITC and 10 wt% UMP.

**Figure 4:**
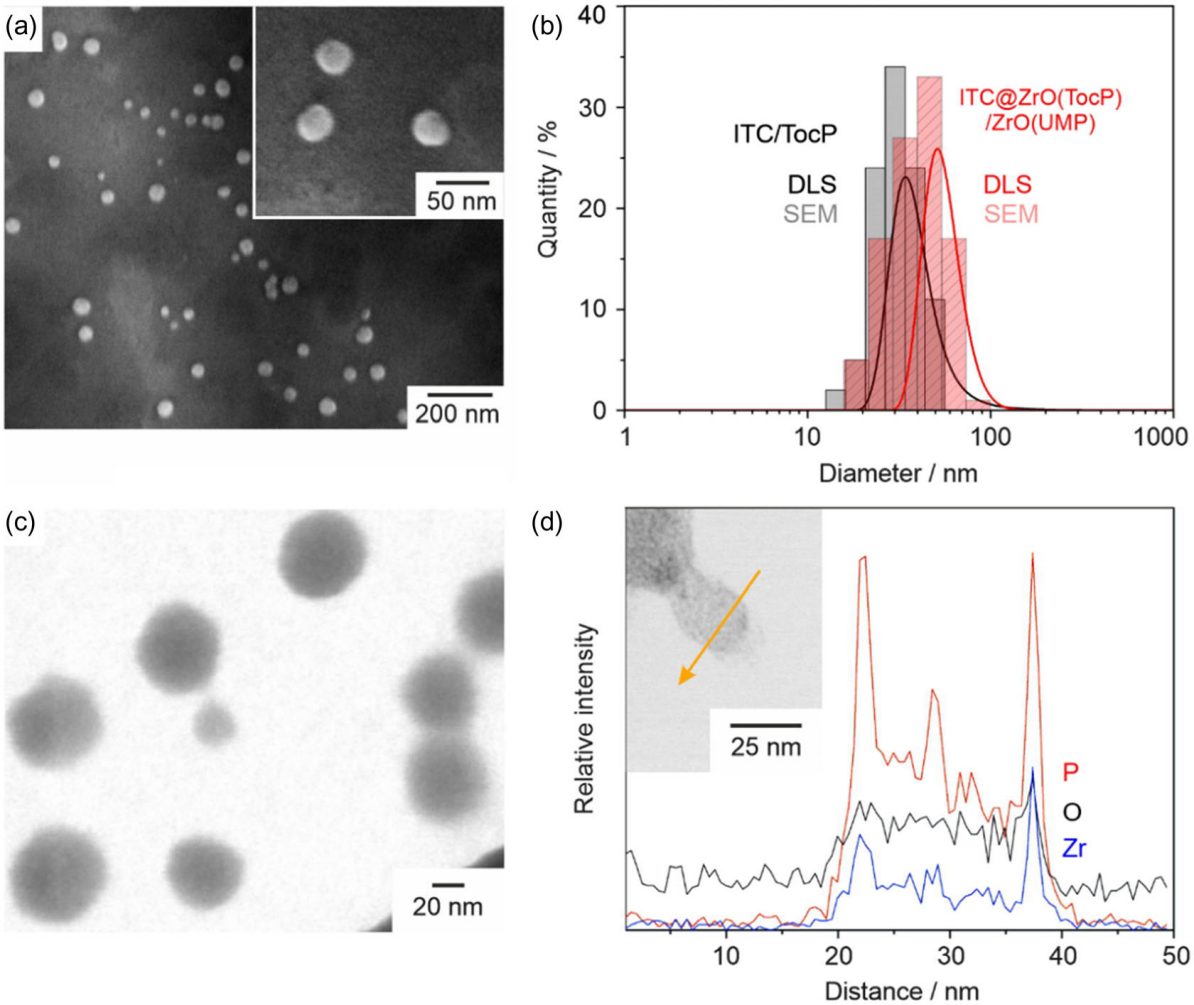
Size and structure of the ITC@ZrO(TocP)/ZrO(UMP) core@shell nanocarriers: a) SEM images with different magnification; b) size distribution of ITC@ZrO(TocP) and of ITC@ZrO(TocP)/ZrO(UMP) core@shell nanocarriers according to DLS (in water) and SEM (statistical evaluation of ≥100 on SEM images); c) STEM image; d) EDXS linescan along yellow line on HAADF‐STEM image (c) showing the elemental distribution of Zr, O, and P.

In addition to the analysis of the chemical composition, the size and structure of the ITC/TocP core nanoparticles and of the ITC@ZrO(TocP)/ZrO(UMP) nanocarriers were investigated by scanning electron microscopy (SEM), dynamic light scattering (DLS), scanning transmission electron microscopy (STEM), and energy‐dispersive X‐ray spectroscopy (EDXS). SEM images show spherical particles with a mean diameter of 31.8 ± 8.2 nm (calculated by statistical evaluation of 100 particles on SEM images; **Figure** **4**a). DLS of aqueous suspensions shows a mean hydrodynamic diameter of 40.1 ± 15.5 nm for the ITC/TocP core nanoparticles (Figure 4b). The successful formation of a shell is validated by the increased size of the ITC@ZrO(TocP)/ZrO(UMP) nanocarriers (DLS: 55.8 ± 13.0 nm; SEM: 41.5 ± 13.9 nm; Figure 4a–c). Moreover, EDXS indicates the presence of the core@shell structure (Figure 4d). Thus, EDXS linescans show the Zr and P concentration to be significantly higher at the surface of the ITC@ZrO(TocP)/ZrO(UMP) nanocarriers. Based on the EDXS linescan, finally, the ITC/TocP particle core can be deduced to have a diameter of about 15 nm, and the ZrO(FdUMP) particle shell can be estimated to a thickness of about 5 nm for a smaller core@shell nanoparticle with a total size of about 25 nm (Figure 4d). Finally, it must be noticed that the nanocarriers are highly sensitive to high‐energy electron bombardment due to their high organic content, so that they show rapid decomposition/evaporation during electron microscopy.

## 3 In Vitro Characterization

For the in vitro characterization, the designation of the nanocarriers is simplified in the following to ITC‐FdUMP‐NC for the ITC@ZrO(TocP)/ZrO(FdUMP) core@shell nanocarriers with two drugs. ITC‐NC refers to ITC@ZrO(TocP)/ZrO(UMP) core@shell nanocarriers with ITC only and FdUMP‐NC to ZrO(TocP)/ZrO(FdUMP) nanocarriers with FdUMP only. Furthermore, REF‐NC indicate drug‐free ZrO(TocP)/ZrO(UMP) nanocarriers as a reference. REF‐ITC‐5FU indicates a reference solution with both dissolved drugs. REF‐ITC and REF‐5FU indicate a solution either containing ITC or 5‐FU only.

### 3.1 Cell Uptake

**Figure 5:**
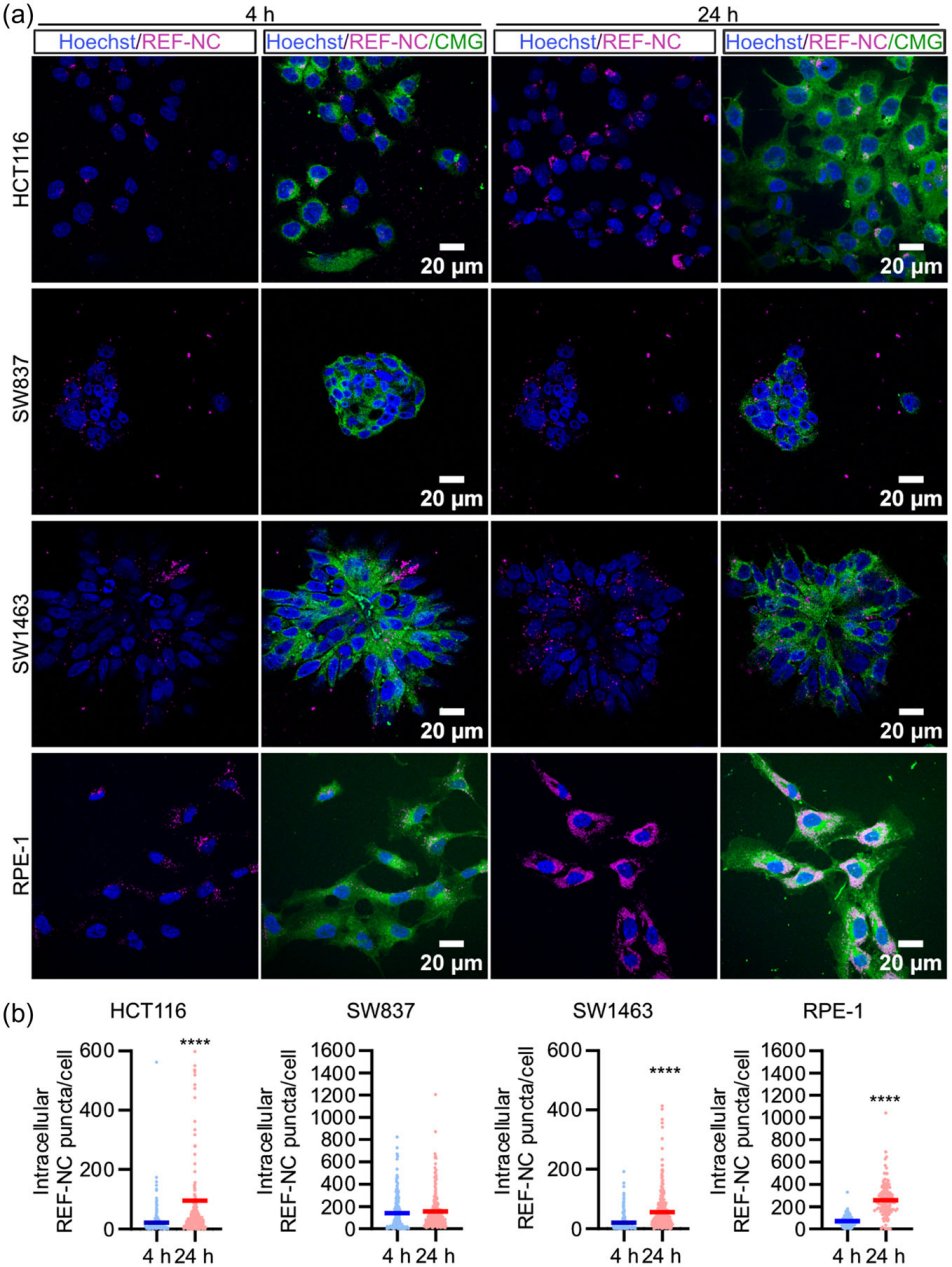
Uptake efficiency of core@shell nanocarriers in different cell lines: a) Representative confocal microscopy images of HCT116, SW837, SW1463 cancer cells and RPE‐1 normal cells treated with 230 μg mL^−1^ of DUT647‐labeled REF‐NC for 4 and 24 h. Cells stained with cell mask green (CMG) nuclei stained with Hoechst 33 342. b) Dot plots of intracellular nanocarrier puncta per cell, analyzed by counting the nanocarrier puncta masked by CMG segmentation from (a) for HCT116, SW837, SW1463, and RPE‐1 cells. Data presented in (b) obtained from two biological replicates; ****p < 0.0001; unpaired two‐tailed student's *t*‐test.

For optimal nanocarrier‐mediated drug delivery, an efficient uptake by the target cells is mandatory. Therefore, the uptake of the drug‐free REF‐NC were first investigated in different CRC cell lines. This includes a human colon cancer cell line HCT116 and the two human rectal cancer cell lines SW1463 and SW837. In addition, RPE‐1 – an immortalized normal human retinal epithelial cell line – was used to compare the uptake efficiency of the nanocarriers (**Figure** **5**a). After exposing the cell lines to DUT647‐labeled REF‐NC (with the fluorescent dye DUT647 located in the nanocarrier shell) (see Figure 3) either for 4 or 24 h, the intracellular fluorescence signals were analyzed by confocal microscopy. Notably, all cell lines, except for the human rectal cancer cell line SW837, display a time‐dependent increase in the intracellular DUT647‐based fluorescence as measured by the number of signal points or puncta per cell (Figure 5a,b). Although no time‐dependent increase of the uptake was observed for SW837, interestingly, the uptake was as efficient as observed in the normal RPE‐1 cell line (Figure 5a,b). Overall, these data show that all cell lines successfully internalize nanocarriers, albeit with different levels of efficiency.

**Figure 6:**
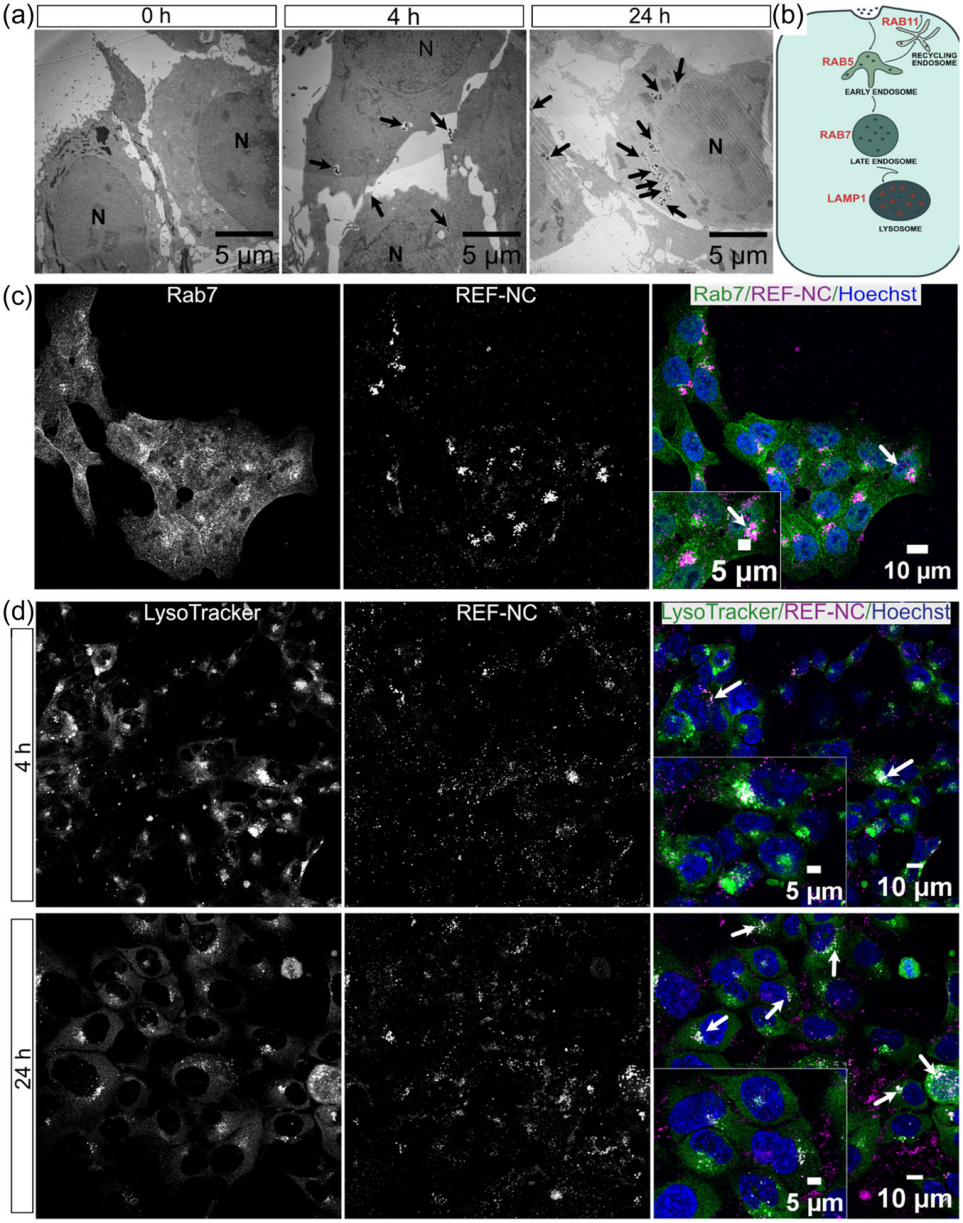
Accumulation of REF‐NCs in acidic vesicular compartments: a) Representative electron microscopy images of HCT116 cells untreated (0 h) or treated with 230 μg mL^−1^ of REF‐NC for 4 or 24 h. Black arrows point to extracellular or intracellular accumulation of REF‐NC. b) Scheme illustrating the classical endosomal trafficking pathway with protein markers serving as classical indicators for each endolysosomal compartment. c) Confocal microscopy images of HCT116 treated with 230 μg mL^−1^ of REF‐NC (middle) for 24 h. Cells immunostained with antibodies against Rab7 and nuclei labeled with the dye Hoechst 33342 merged). Arrows in the magnified inset of the merged images (right) indicate colocalization (white) between REF‐NC (magenta) and Rab7 (green) in the merged image. d) Representative confocal microscopy images of HCT116 cells treated with 230 μg mL^−1^ of REF‐NC labeled with DUT647 for 4 or 24 h showing intracellular acidic compartments labeled by LysoTracker (left) and nuclei stained with Hoechst 33342 (middle). White arrows point to colocalization (white) between LysoTracker (green) and REF‐NC (magenta) in the merged images (right).

After proving the efficient uptake of REF‐NC in different cell lines, we investigated the subsequent intracellular trafficking to the subcellular compartments in detail by transmission electron microscopy (TEM) after exposing HCT116 cells to DUT647‐labeled REF‐NC for 4 or 24 h (**Figure** **6**a). Here, the presence of zirconium in the nanocarriers is beneficial since such a heavy element is absent in cells. Hence, its strong electron absorbance is indicative for the localization of the nanocarriers. Interestingly, REF‐NCs are found after 4 h extracellularly around the cell surface or enclosed intracellularly in membrane‐bound organelles, mostly at the cell periphery (Figure 6a). In contrast, most of the REF‐NCs are taken up intracellularly and all membrane‐bound organelles containing nanocarriers are clustered around the perinuclear area after 24 h of incubation (Figure 6a). This finding agrees with immunofluorescence (IF) images after 24 h of incubation (Figure 5a) and suggests REF‐NCs to be endocytosed and indicates that they reach perinuclear organelles like late endosomes or lysosomes via early endosomes. This also aligns with previous TEM studies indicating the nanocarriers to be internalized by macrophages within the initial 10 min, followed by localization within large vesicles in the cells.^[^ ^20^ ^]^ Since the nanocarriers are internalized and trafficked intracellularly within membrane‐bound compartments that eventually converge at perinuclear locations resembling endolysosomal compartments, our next step involved examining the colocalization of the nanocarriers with various classical endosomal compartment markers (Figure 6b). After 24 h of REF‐NC incubation, HCT116 cells show no colocalization of REF‐NC with both the early endosomal marker Rab5 and the recycling endosomal marker Rab11 (Figure S6a, Supporting Information: upper and middle panel). As illustrated in Figure 6b, this result suggests that the REF‐NC may have traversed early endosomal compartments already after 24 h, reaching late endosomes or lysosomes where they are likely to be disintegrated. As expected, REF‐NC reaching the perinuclear compartments at this incubation time were positive for the late endosomal marker Rab7 (Figure 6c). This implies that they are transported toward acidic organelles such as late endosomes (Figure 6b). In agreement with TEM results, the REF‐NC accumulate after 24 h of incubation at the perinuclear area colocalizing with the lysosomal marker protein LAMP1 (Figure S6a, Supporting Information, lower panel). This suggests that REF‐NC are trafficked toward the endolysosomal compartments.

In alignment with these results, REF‐NC observed in the perinuclear regions show a strong colocalization with the LysoTracker dye, which specifically labels acidic organelles like the late endosomes and lysosomes (Figure 6d). Additionally, the REF‐NC show an increased colocalization with LysoTracker when compared to the short time exposure of 4 h as higher amounts of the REF‐NC reach the perinuclear acidic compartments after 24 h of exposure (Figure 6d). Overall, these findings reveal for the first time how core@shell nanocarriers are transported through membrane‐bound organelles, ultimately reaching the late endosomes and lysosomes. Notably, these compartments exhibit partial positivity for the late endosomal marker Rab7 and the lysosomal marker LAMP1. With their capability to be fluorescently labeled for intracellular tracking, enhancing their utility in monitoring delivery processes, core@shell nanocarriers generally possess a highly multifunctional feature for drug delivery.

### 3.2 In Vitro Efficacy of FOLFIRI‐Type Nanocarriers

A cocktail of chemotherapeutics, folinic acid, and 5‐fluorouracil (5‐FU) either with oxaliplatin (FOLFOX) or with ITC (FOLFIRI) is frequently given to CRC patients either as neoadjuvant or adjuvant therapy, usually in conjunction with available targeted therapies. Despite different advancements, the efficacy of a 5‐FU‐based chemotherapy still remains suboptimal, with many patients experiencing severe side‐effects, disease progression, or recurrence.

Therefore, this study utilizes core@shell nanocarriers with high chemotherapeutic drug payload for direct delivery of the poorly soluble drug ITC and the active drug FdUMP, mimicking the FOLFIRI chemotherapy regime. To assess their efficacy, we analyzed the cytotoxic effectiveness of ITC‐FdUMP‐NC with ITC as the nanocarrier core and FdUMP in the nanocarrier shell. For comparison, nanocarriers consisting only of a single drug (i.e., ITC‐NC with ITC only, FdUMP‐NC with FdUMP only) were included in the cell‐based assays as well. Moreover, the free drugs in combination (REF‐ITC‐5FU) or as single drugs (REF‐ITC, REF‐5FU) were applied as solutions. The cytotoxic efficacy of the free drugs and the nanocarriers was assessed using a CellTiterGlo assay, measuring the cell viability after 72 h, 5 days (RPE‐1) or 7 days (CRC cell lines) in response to the treatment with varying concentrations of ITC (20, 50, 80, 100 μm). Due to the rapid proliferation rate of the RPE‐1 cells, which leads to early confluence in untreated control cells, the cell viability was here assessed 5 days after treatment. Importantly, the nanocarriers contain twice as much ITC as FdUMP. This means that for all given concentrations of ITC, the concentration of FdUMP/5‐FU is half of the ITC concentration (i.e., 10, 25, 40, and 50 μm).

For a reliable result of the cytotoxicity assays, a complete set of controls for evaluating the cytotoxic efficacy between the nanocarriers and the freely dissolved drugs was used. To this concern, free ITC was dissolved in DMSO and free 5‐FU in water. Consequently, DMSO was also included as vehicle control to ensure that any observed effect was not due to the solvent itself. REF‐NC serve as a drug‐free control for the nanocarriers, allowing to examine their characteristics in absence of an active chemotherapeutic drug. Additionally, TocP in the same concentration as included as surfactant in the nanocarriers was included in the assay (REF‐TocP), providing insights into its potential influence on the experimental outcomes. The relative viability percentage was measured by normalizing the viability values of each treatment to the untreated conditions.

**Figure 7:**
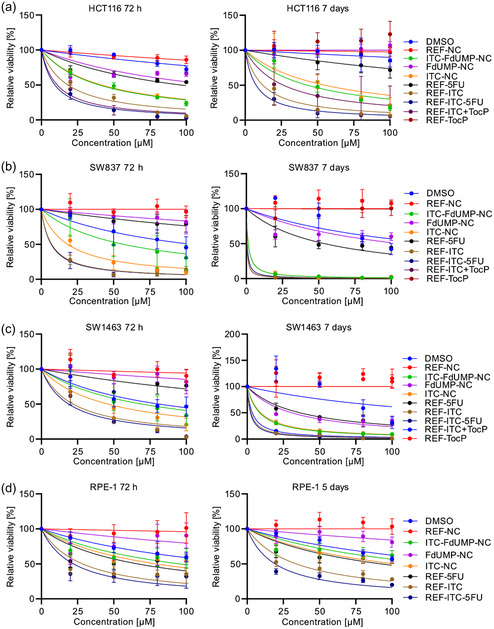
In vitro efficacy of FOLFIRI‐NC, ITC‐FdUMP‐NC in CRC cell lines: a–d) Dose‐response curves of CRC cell lines HCT116 (a), SW387 (b), SW1463 (c), and normal RPE‐1 cells (d), treated with a range of ITC concentrations (20, 50, 80, 100 μm) and 5‐FU concentrations (10, 25, 40, 50 μm) either in NCs (ITC‐FdUMP‐NC, ITC‐NC, FdUMP‐NC) or as freely dissolved drugs (REF‐ITC/5FU, REF‐ITC, REF‐5FU). Effects of REF‐ITC‐TocP and REF‐TocP also analyzed in HCT116, SW837, and SW1463 cells. Dose‐response curves generated by measuring the relative cell viability after 72 h, 5 days (RPE‐1) or 7 days (CRC cell lines) of treatment at the indicated drug concentration. DMSO used as a control vehicle for freely dissolved ITC; drug‐free REF‐NC are included as a negative control. Data from (a–d) presented as mean ± SD of three biological replicates with two technical replicates; IC_50_ values of each treatment calculated by fitting the dose‐response data from 5 days (RPE‐1) or 7 days (CRC cell lines) of treatment to a non‐linear regression curve (Table 1).

Unlike REF‐NC and REF‐TocP, which both show no effect, DMSO reduces the cell viability in a concentration‐dependent manner in all cell lines at both time points (**Figure** **7**a–d). All treatments reduce the viability of the CRC cells in a drug concentration‐dependent manner at both time points, albeit to different extents. Across all cell lines, FdUMP‐NC and REF‐5FU reduce the cell viability less than ITC, thus, proving ITC to be more cytotoxic than FdUMP/5FU (Figure 7a–d). Interestingly, the two rectal cancer cell lines are more sensitive to the FOLFIRI chemotherapy than HCT116 and RPE‐1, as both ITC only and the ITC/5FU combination therapy show a lower dose cytotoxic response in SW837 and SW1463 for the free drugs (REF‐ITC and REF‐ITC‐5FU) as well as for the nanocarriers (ITC‐NC and ITC‐FdUMP‐NC) (Figure 7b,c). Free drugs exhibit greater cytotoxicity effects than all nanocarriers across all the cell lines tested after 72 h of treatment (Figure 7a–d: right graphs). Interestingly, ITC‐FdUMP‐NC and ITC‐NC display cytotoxicity effects comparable to their free drug counterparts in both rectal cancer cell lines after 7 days of treatment. This could suggest a certain delay of the nanocarrier effect in comparison to the free drugs (Figure 7b,c: left graphs). In contrast to the rectal cancer cell lines, the free drugs are more potent than the nanocarriers in HCT116 and normal RPE‐1 cells even after 7 and 5 days of treatment, respectively (Figure 7a,d: right graphs).

Altogether, the IC_50_ values for the nanocarriers are found to be higher than those of the free drugs across all cell lines (**Table** **1**), as measured after 7 days (CRC cell lines) and 5 days (RPE‐1 cell line) of treatment. However, the differences are much smaller in the rectal cancer cell lines. Of note, it is impossible to calculate IC_50_ values for the ITC/5‐FU combination treatment – for both the free drugs and the nanocarriers. Therefore, the IC_50_ values for the combination treatment are calculated against the concentrations of both individual drugs (Table 1). Both rectal cell lines SW837 and SW1463 are more sensitive to the cytotoxicity of the nanocarriers than HCT116 and normal RPE‐1 cell lines. Interestingly, across all cell lines, the uptake efficiency of REF‐NC (Figure 5) does not correlate with the extent of the cytotoxic effects induced by the drug‐containing nanocarriers. Despite displaying a lower nanocarrier uptake rate than observed in HCT116 and normal RPE‐1 cells (Figure 5), SW1463 cells show higher cytotoxic effects in response to the nanocarriers. Although the normal RPE‐1 cell line displays the highest uptake rates, it paradoxically shows only small cytotoxic effects not only in response to the nanocarriers but also to the free drugs. This suggests the ability of normal, non‐transformed cells, which have their intact metabolic status and DNA damage‐repair machinery, to circumvent the cytotoxic effects exerted by chemotherapeutic drugs (Table 1).

**Table 1:** IC_50_ values of the nanocarriers in comparison to values of the freely dissolved drugs (single or in combination) after 5 days in RPE‐1 cells or 7 days in all other cell lines.

Treatment	IC_50_ [μm]			
	HCT116	SW837	SW1463	RPE‐1
ITC‐FdUMP‐NC	20/39.9	0.7/1.4	4.3/8.6	65.1/130.2
FdUMP‐NC	119.8	105.4	30.8	524.3
ITC‐NC	41.5	1.4	9.4	101.6
REF‐5FU	95.9	54.0	36.5	92.3
REF‐ITC	18.3	0.5	2.7	33.8
REF‐ITC‐5FU	4.7/9.3	0.3/0.6	1.2/2.3	9.6/19.2

As the core@shell nanocarriers employ a high chemotherapeutic drug payload and a direct delivery mechanism for the poorly soluble drug ITC and the active drug FdUMP, they may also play a crucial role in eliminating the need for active transporters such as the human nucleoside transporters (hENT1 and hENT2) or the organic anionic transporter 2 (hOAT2), which specifically facilitate the cellular uptake of 5‐FU. Reduction in the expression of these transporters is associated with 5‐FU resistance, making nanocarriers a promising tool to circumvent this resistance mechanism. Furthermore, the direct delivery of the active drug FdUMP bypasses the need for additional enzymatic conversion of the prodrug 5‐FU into FdUMP, which addresses potential resistance mechanisms such as an increased expression of enzymes like orotate phosphoribosyltransferase (OPRT), thymidine kinase (TK), and ribonucleotide reductase (RR), which are involved in the conversion of the prodrug 5‐FU into the active drug FdUMP. Although less cytotoxic than the free drugs in vitro, the promising anti‐tumor activity of ITC‐FdUMP‐NC and ITC‐NC suggests the potential for in vivo applications involving a combination of two cytotoxic drugs encapsulated within a single nanocarrier. Such multidrug formulation via nanocarriers offers the option to protect drugs from rapid degradation and metabolism under physiological conditions. In vivo, we expect that the nanocarrier formulation, even when not as potent as the free drugs in vitro, has the advantage of a direct drug delivery to the tumor site as well as a shielding effect provided by the nanocarriers to minimize known side effects (e.g., nausea, vomiting, hepatotoxicity, diarrhea), which are especially critical in elderly cancer patients.

Interestingly, the cytotoxic effects of the nanocarrier appear to be cell‐type‐specific with rectal cancer cells demonstrating a high sensitivity to FOLFIRI‐like ITC‐FdUMP‐NC, which is comparable to the free ITC + 5FU drug cocktail but less toxic to normal RPE‐1 cells. In a similar manner, previous studies showed that, for instance, a nanoparticulate delivery form of cisplatin may outperform the free drug in a human ovarian cancer cell line.^[^ ^21^ ^]^ Moreover, gemcitabine‐loaded nanocontainers show a higher efficacy in pancreatic cancer cell lines in vivo than freely dissolved gemcitabine.^[^ ^14^ ^]^ Our study confirms these findings, showing a slightly greater cytotoxic effect of the freely dissolved drugs than their nanoparticulate counterparts in a 2D cell culture system. However, the extent of this difference varies among different cell lines.

**Figure 8:**
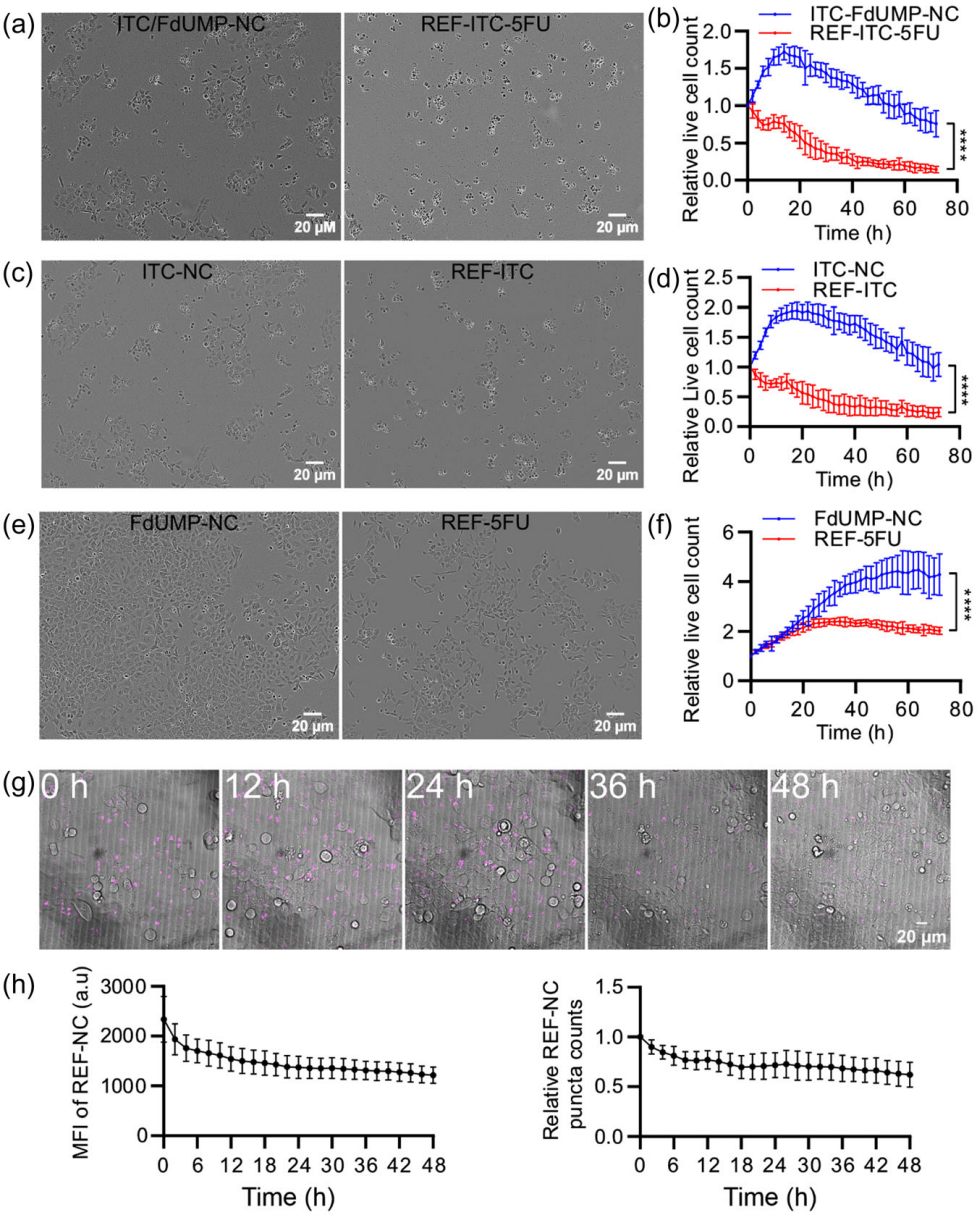
Delayed cytotoxic effect of FOLFIRI‐type ITC‐FdUMP‐NC in HCT116 cells: a) Representative incucyte images of colon cancer HCT116 cells treated with indicated ITC‐FdUMP‐NC and the free‐drug controls REF‐ITC‐5FU and REF‐5FU for 72 h. b) Proliferation kinetics of HCT116 cells treated with ITC‐FdUMP‐NC or REF‐ITC‐5FU measured by calculating the number of live cells at 2‐hour intervals until 72 h. c) Representative incucyte images of HCT116 cells treated with indicated ITC‐NC and the free drug control REF‐ITC for 72 h. d) Proliferation kinetics of HCT116 cells treated with ITC‐NC or REF‐ITC presented by calculating the number of live cells at 2 h intervals until 72 h. e) Representative incucyte images of HCT116 cells treated with the indicated FdUMP‐NC and the drug‐free control REF‐5FU for 72 h. f) Proliferation kinetics of HCT116 cells treated with FdUMP‐NC or REF‐5FU measured by calculating the number of live cells at 2 h‐ intervals until 72 h. (b,d,e) Relative live‐cell count measured by normalizing the cell count of untreated conditions. g) Time‐lapsed (12 h interval) confocal images of HCT116 cells (brightfield) treated with 57.5 μg mL^−1^ of DUT647‐labeled REF‐NC (magenta) until 48 h. h) Mean fluorescence intensity (MFI) of REF‐NC and relative REF‐NC puncta per image from (g) analyzed by Imaris software; scale bars in images (g) represent 20 μm.

To further evaluate the differences in the efficacy of the free drugs and the FOLFIRI‐type nanocarriers, real‐time cell‐live living imaging of HCT116 cells was performed, enabling the analysis of cell proliferation kinetics in response to various treatment conditions. Accordingly, HCT116 cells treated with ITC‐FdUMP‐NC and their corresponding free‐drug counterparts exhibit significantly different proliferation kinetics as measured by live‐cell counts over time (**Figure** **8**a–f). In contrast to the freely dissolved drugs, in which live‐cell counts decreased immediately after treatment at 2 h, ITC‐FdUMP‐NC (Figure 8a,b) and ITC‐NC (Figure 8c,d) reduce the live‐cell counts at much later time points (after 12–18 h) with the cell counts increasing for some initial time points. Interestingly, both FdUMP‐NC and free FdUMP plateaued in live‐cell count over time after different time points, indicating 5‐FU‐induced cell division arrest, albeit with the effects of the drugs manifesting at different time points (Figure 8e,f). In addition to the cytostatic effects of FdUMP/5‐FU in HCT116, this also suggests that both the cytotoxic and cytostatic effects of the nanocarriers are delayed in comparison to the freely dissolved drugs. This delayed cytotoxic effect can be attributed to the distinct spatial and temporal regulations via the endocytic and trafficking pathway post‐cellular uptake. Nanocarrier‐encapsulated chemotherapeutic drugs undergo a unique uptake and intracellular trafficking pathway, distinct from free drugs like 5‐FU and ITC, which are transported or diffused inside the cell for enzymatic conversion into active drugs. Understanding this cellular trafficking pathway enables the addition of modulating molecules to the nanocarriers and enhances the drug efficacy. For instance, incorporating endolysosomal membrane‐disrupting agents like chloroquine in the nanocarriers could potentially increase the drug release.^[^ ^22^ ^]^

To further investigate the fate of internalized nanocarriers over time, we analyzed zirconium‐based nanocarriers by TEM at 6, 24, and 96 h of incubation (Figure S6b, Supporting Information). Initially, at 6 h, the nanocarriers are near the cell surface. After 24, 48, 72, and 96 h, they accumulate in cellular compartments within the perinuclear area, whereas no nanocarriers were observed in the cytosol as indicated by the lack of a zirconium signal in the cytosol on TEM images even after 96 h (Figure S6b, Supporting Information, TEM data after 48 and 72 h not shown). Based on these data and the observed colocalization of the perinuclear REF‐NC with Rab7 and LAMP1 (Figure 6c and S6a, Supporting Information), we propose that the nanocarriers are sequestered in the endolysosomal compartments, where they slowly disintegrate, releasing the drugs into the cytosol. In fact, this drug release into the cytosol is supported by the delayed cytotoxic effects of ITC and FdUMP delivered by the nanocarriers (Figures 7 and 8a–f).

To indirectly analyze how fast the nanocarriers degrade with release of the respective drug, the decay of the intracellular fluorescence intensity of REF‐NC was examined over time in HCT116 cells for 48 h (Figure 8g). To exclude a continuous uptake and to track only the internalized nanocarrier for degradation, the residual REF‐NC in the media were completely removed after 24 h of incubation. As expected, both the relative mean fluorescence intensity (MFI) of REF‐NC as well as the relative REF‐NC puncta counts show a gradual decrease over time with around 40 % reduction at the 48‐hour time point, thus, implying a slow time‐dependent degradation of the nanocarriers in the cells for drug release (Figure 8h). This indicates that – although the free drugs exhibit a more efficient cytotoxic effect – the chemotherapeutics transported via our core@shell nanocarriers exhibit different pharmacodynamics with delayed cytotoxic and/or cytostatic effects compared to free drugs, which can lead to a more sustained drug release. Such delayed cytotoxic response associated with the core@shell nanocarriers can potentially enhance the therapeutic outcomes by maintaining effective drug concentrations over an extended period of time. In summary, both ITC‐FdUMP‐NCs and ITC‐NC show a promising anti‐tumor activity in conventional 2D cell culture systems. The nanocarriers display a slower yet sustained drug‐release profile, which could offer advantages over the rapid action of conventional freely dissolved drugs, especially in resistant cell lines.

### 3.3 In Vitro Efficacy of FOLFIRI‐Type Nanocarriers in Rectal Cancer Patient‐Derived Organoids

Since FOLFIRI‐type ITC‐FdUMP‐NC show a distinct cytotoxic efficacy, especially in the two rectal cancer cell lines, their effect was further evaluated in patient‐derived organoids (PDOs) developed from human rectal adenocarcinoma as a more complex in vitro system. PDOs recapitulate both the morphological and the genetic features of the original tumors from the patients. Most importantly, post hoc studies indicate that PDOs may mirror the clinical response of individual patients to therapy.^[^ ^23^ ^]^

**Figure 9:**
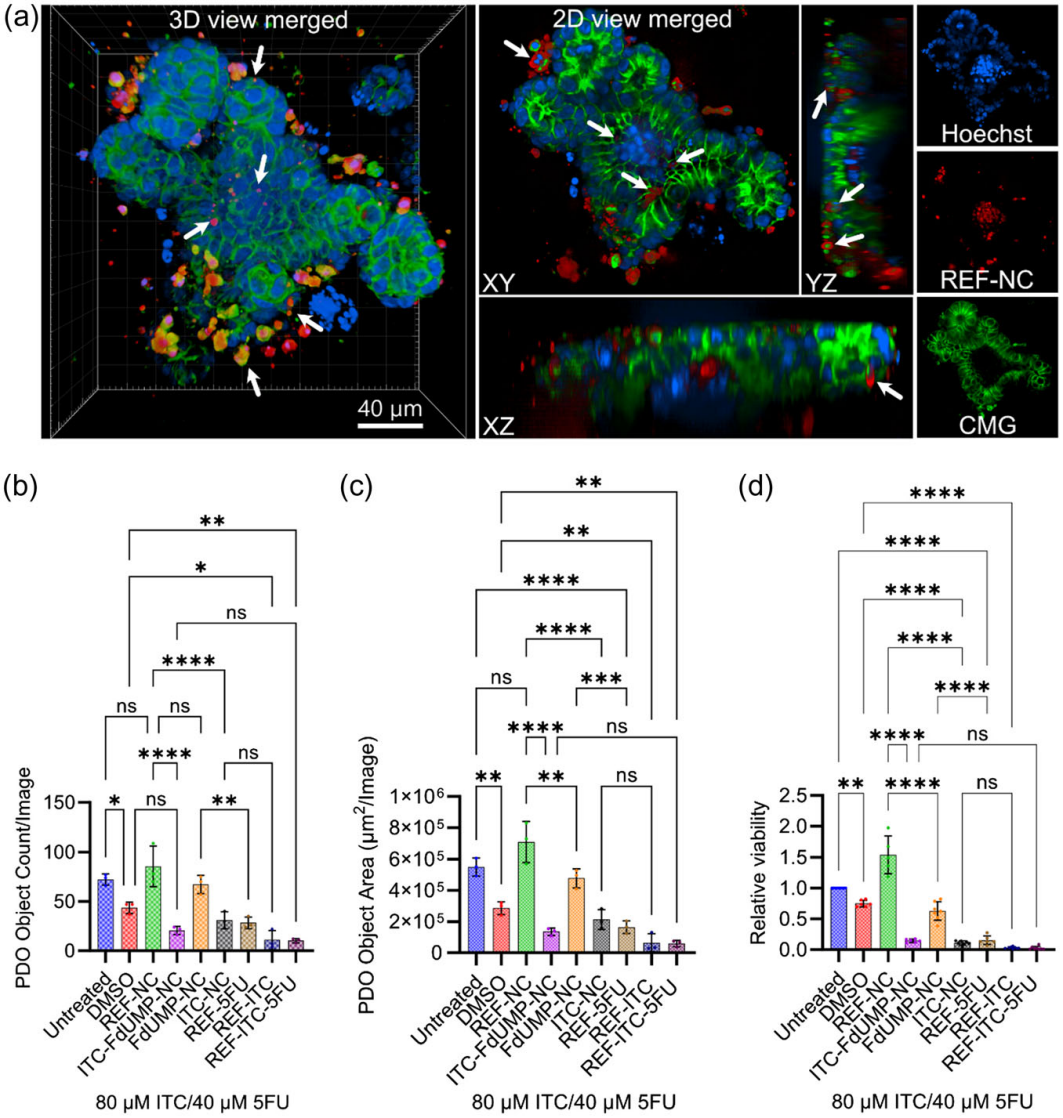
Uptake of REF‐NC and potency of FOLFIRI‐type ITC‐FdUMP‐NC and the free drugs in rectal cancer patient‐derived organoids (PDO): a) representative 3D and 2D (*xy*‐, *yz*‐, *xz*‐view) spinning disk confocal images of PDOs, grown in a media with 10% Matrigel v/v and treated with 230 μg mL^−1^ DUT647‐labeled REF‐NC for 24 h. PDOs stained with cell mask green (CMG) and Hoechst 33342 (blue) for nuclei. White arrows point to DUT647‐labeled REF‐NC (red) signals found in some cells at the periphery or in the lumen of the PDO. Yellow represents colocalization between CMG (green) and DUT647 (red). b,c) Quantification of incucyte images (Figure S7, Supporting Information) obtained after 5 days of treatment by using the inbuilt incucyte analysis software showing PDO object count per image (b) and PDO object area per image (c). d) Viability assays of rectal cancer PDOs treated with the indicated controls, NCs, and free drugs (single drugs and combination) for 5 days. Relative viability measured by normalizing the value of untreated conditions. Data from (b–d) presented as mean ± SD of three biological replicates; *p < 0.01, **p < 0.001, ****p < 0.0001, *****p < 0.00001, ns = non‐significant; ordinary one‐way ANOVA followed by Tukey's multiple comparisons test.

Based on these results demonstrating a higher efficacy of the nanocarriers against rectal cancer cell lines than the colon HCT116 cells (Table 1), PDOs from a rectum tumor were used to analyze the cytotoxicity efficacy of nanocarriers. Firstly, the uptake of REF‐NC within PDOs was analyzed by using spinning disk confocal microscopy. To delineate the cells, we used cell mask green (CMG) a plasma‐membrane stain. Notably, tumor cells within the PDOs exhibit a good uptake of nanocarriers, as evidenced by the presence of REF‐NC labeled with fluorophore DUT647 (red) within the cells. Most importantly, the REF‐NC uptake can be detected in tumor cells within the PDO (in both villi and crypt‐like domains) across different *xy*‐, *yz*‐, and *xz*‐planes (**Figure** **9**a). These findings strongly suggest that nanocarriers are capable to penetrate the complex 3D structures of PDOs to reach tumor cells, underscoring their potential as drug delivery vehicles in this context. Subsequently, PDOs were treated either with FOLFIRI‐type ITC‐FdUMP‐NC (with 80 μm of ITC and 40 μm of FdUMP) or with the corresponding free drugs ITC and 5‐FU to further elucidate the cytotoxicity efficacy of the nanocarriers. To analyze the growth and morphology in response to the different treatment conditions, PDO images acquired with an incucyte cell‐live‐imaging system were assessed during 5 days of treatment. Specifically, the PDO growth and morphology were analyzed by measuring the PDO count per image and the PDO area per image. Unlike controls (untreated, DMSO, REF‐NC), where organoids expand with normal morphology, and except for FdUMP‐NC, both ITC‐FdUMP‐NC and ITC‐NC and the free drug treatments induce cell death, where no PDOs were visible in the images acquired by incucyte after 5 days (Figure S4, Supporting Information). The quantificationsC of the images show that all treatment conditions significantly reduce both PDO counts and PDO area per image in comparison to the respective controls (Figure 9b,c). As in the 2D cell culture (Figure 7), ITC again turned out to be more cytotoxic than FdUMP/5‐FU in PDO as evidenced by FdUMP‐NC showing the least effect followed by the free‐drug 5‐FU. In alignment with the observations with the rectal cancer cell lines (Figure 9b,c), the FOLFIRI‐type ITC‐FdUMP‐NC are equally cytotoxic as the free‐drug counterparts ITC + 5‐FU.

To further validate the cell‐live‐imaging data assessing cytotoxicity over time, a CellTiterGlo assay was performed to measure the relative PDO viability after 5 days of treatment (Figure 9d). In complete alignment with the incucyte results (Figure 9a–c), all nanocarriers and free‐drug treatments significantly reduce the relative PDO viability. Again, FdUMP‐NC show the least toxic effect. The ITC‐FdUMP‐NC and the ITC‐NC as well as the free‐drugs ITC + FdUMP and ITC‐only treatments reduce the cell viability with similar behavior of nanocarriers and free drugs (Figure 9d). Taken together, ITC‐FdUMP‐NC exhibit a strong cytotoxic potency similar to the free drug chemotherapeutics in rectal cancer PDOs, demonstrating that they represent an attractive model to assess the efficacy of core@shell nanocarriers in the era of personalized medicine in the future.

## 4 Conclusion

ITC@ZrO(TocP)/ZrO(FdUMP) core@shell nanocarriers (briefly designated ITC‐FdUMP‐NC) containing the clinically relevant chemotherapeutics irinotecan (ITC) and fluoro‐2′‐deoxyuridine‐5′‐phosphate (FdUMP) (active derivative of 5′‐fluoruracil/5‐FU) are prepared and examined as a new type of nanocarrier for the first time. The ITC‐FdUMP‐NC exhibit a high drug payload with 22% of ITC in the particle core and 10% of FdUMP in the particle shell. This drug combination matches with FOLFIRI (combination of dissolved free drugs ITC and 5‐FU), representing the standard second‐line chemotherapy regime for patients with metastatic colorectal cancer (CRC), and is first shown with high drug payload in a single nanocarrier. A specific challenge of the synthesis is to combine lipophilic ITC and hydrophilic FdUMP in a single, water‐insoluble nanocarrier. To this concern, we used a combination of a solvent‐antisolvent strategy to obtain ITC nanoparticles, which become colloidally stable and dispersible in water by a ZrO(TocP)/ZrO(FdUMP) shell (TocP: tocopherolphosphate, a derivative of vitamin E). The ITC@ZrO(TocP)/ZrO(FdUMP) core@shell nanocarriers are available in water with a mean diameter of 41.5 ± 13.9 nm and a particle concentration of 13 mg mL^−1^ (with 2.9 mg mL^−1^ ITC and 1.3 mg mL^−1^ of FdUMP).

Fluorescence labeling allows to assess efficient tumor‐cell uptake of ITC‐FdUMP‐NC over time in different cell lines (human colon cancer cell lines HCT116 and HT29, human rectal cancer cell lines SW1463 and SW837, immortalized normal human retinal epithelial cell line RPE‐1). For the first time, the distinct trafficking pathway of the nanocarriers through membrane‐bound organelles, reaching acidic compartments such as lysosomes and endosomes is unraveled. Specifically, the nanocarriers are transported toward late endosomes and lysosomes as demonstrated by their colocalization with Rab7 and LAMP1. While it is evident that the cytotoxic efficacy of nanocarrier‐delivered ITC and FdUMP results from their release from endosomes/lysosomes into the cytosol, the mechanisms facilitating endosomal escape for core@shell nanocarriers or the release of free ITC or FdUMP from endosomal vesicles needs further exploration. Elucidating the complete membrane‐trafficking pathway responsible for the nanocarrier‐mediated drug release into the cytosol is of high importance and offers further options to incorporate agents to enhance the drug efficacy and/or to inhibit drug efflux pumps. Unlike the free drugs, which are rapidly flushed out upon cellular entry by known protein pumps, altered drug subcellular localization upon delivery through nanocarriers may also circumvent common ITC and/or 5‐FU drug resistance mechanisms mediated by efflux pumps on the plasma membrane.

Specifically, the FOLFIRI‐type ITC‐FdUMP‐NC   show an efficacy comparable to the free drugs in the human rectal cancer cell lines. They also demonstrate effective drug delivery even within rectal cancer patient‐derived organoids (PDOs) as more complex 3D structures. The observed delayed cytotoxic and/or cytostatic effects of the core@shell nanocarriers in CRC cell cultures compared to free drugs might even improve the therapeutic outcome by maintaining effective drug concentrations over an extended period. In regard of the varying potencies of ITC and FdUMP/5‐FU, in principle, adjustments in the dosage of each drug are possible as needed for the best treatment efficacy. Importantly, FdUMP‐ITC‐NC and ITC‐NC are as effective as their free drug counterparts in the 3D organoid culture, which more closely mimics in vivo conditions. These results support the potential of an adaptable core@shell drug delivery system and motivate their use in further in vivo experiments for future preclinical studies. Core@shell nanocarriers can offer a compelling avenue for advancing drug delivery in cancer therapy. Their unique structure allows for precise control over the drug loading for optimal therapeutic concentrations at the target site. Moreover, these nanocarriers can be functionalized with antibodies for specific tumor targeting and labeled for tracing their distribution within the body. Importantly, they exhibit toxicity profiles similar to conventional free drugs, indicating their efficacy. By harnessing these properties, core@shell nanocarriers hold great promise for improving chemotherapy outcomes while mitigating the severe side effects of conventional FOLFIRI chemotherapy for patients with metastatic CRC.

## Conflict of Interest

The authors declare no conflict of interest.

## Author Contributions

**Silke Notter**: Formal analysis (equal); Investigation (equal); Validation (equal); Writing—original draft (equal). **Dolma Choezom**: Formal analysis (equal); Investigation (equal); Validation (equal); Writing—original draft (equal). **Titus Griebel**: Investigation (supporting). **Fernanda Ramos‐Gomes**: Investigation (equal); Validation (equal). **Wiebke Möbius**: Data curation (supporting); Formal analysis (supporting); Methodology (supporting). **Tiago De Oliveira**: Data curation (supporting); Formal analysis (supporting). **Lena‐Christin Conradi**: Conceptualization (equal); Methodology (equal). **Frauke Alves**: Conceptualization (equal); Formal analysis (equal); Funding acquisition (equal); Investigation (equal); Project administration (equal); Supervision (equal); Writing—review & editing (equal). **Claus Feldmann**: Conceptualization (equal); Funding acquisition (equal); Project administration (equal); Resources (equal); Writing—review & editing (equal).

## Supporting information

Supplementary Material

## Data Availability

The data that support the findings of this study are available from the corresponding author upon reasonable request.
