# Supplementary material for: High‐Load Core@Shell Nanocarriers with Irinotecan and 5‐Fluorouracil for Combination Chemotherapy in Colorectal Cancer
Source: Small Sci. 2024 Aug 19;4(11):2400196. doi: 10.1002/smsc.202400196 (PMC11935085; doi:10.1002/smsc.202400196)
Supplement: Supplementary file 1 — Supplementary Material [file SMSC-4-2400196-s001.pdf]

Supporting Information

High-Load Core@Shell Nanocarriers with Irinotecan and 5-Fluorouracil for Combination Chemotherapy in Colorectal Cancer

Silke Notter[1], Dolma Choezom[2,3,4], Titus Griebel[2,3], Fernanda Ramos-Gomes[3], Wiebke Möbius[3], Tiago De Oliveira[4], Lena-Christin Conradi[4], Frauke Alves[2,3,5] and Claus Feldmann[1]**

[1] Institute of Inorganic Chemistry, Karlsruhe Institute of Technology (KIT), Engesserstrasse 15, 76131 Karlsruhe, Germany
claus.feldmann@kit.edu; Tel.: +49-721-608-42856

[2] University Medical Center Goettingen (UMG), Clinic for Haematology and Medical Oncology, Robert Koch Str. 40, 37075 Goettingen, Germany.

[3] Max-Planck-Institute for Multidisciplinary Sciences (MPI-NAT), Department of Neurogenetics, City Campus, Hermann-Rein-Strasse 3, 37075 Goettingen, Germany
falves@gwdg.de; Tel.: +49-551-20131655

[4] University Medical Center Goettingen (UMG), Department of General, Visceral and Pediatric Surgery, Robert-Koch-Str. 40, 37075, Göttingen, Germany.

[5] University Medical Center Goettingen (UMG), Institute for Diagnostic and Interventional Radiology, Robert Koch Str. 40, 37075 Goettingen, Germany.

<u>**Content**</u>

1. Analytical Tools
2. Synthesis of Core@Shell Nanocarriers
3. Materials Characterization of Core@Shell Nanocarriers
4. *In vitro* Characterization
5. References

1. Analytical Tools

Scanning electron microscopy (SEM). Scanning electron microscopy (SEM) was carried out with a Zeiss Supra 40 VP microscope (Zeiss, Germany), equipped with a Schottky field emitter (2.0 nm resolution). To this concern, diluted aqueous suspensions of the respective nanocarriers were deposited on silicon wafers and left for drying overnight. The acceleration voltage was 5 kV and the working distance was 2-3 mm. Average particle diameters were calculated by statistical evaluation of at least 100 nanoparticles (ImageJ 1.47v software).

Transmission electron microscopy (TEM). Transmission electron microscopy (TEM) and high-angle annular dark-field scanning transmission electron microscopy (HAADF-STEM) were conducted with a FEI Osiris microscope at 200 kV (FEI, The Netherlands). TEM samples were prepared by evaporating aqueous suspensions of the respective nanocarriers on amorphous carbon (Lacey-)film suspended on copper grids.

Energy-dispersive X-ray (EDX) spectroscopy. High-resolution EDXS was performed to analyze the chemical composition of single nanocarriers. The spectra were obtained at 200 kV electron energy with a FEI Osiris microscope that was equipped with a Bruker Quantax system (XFlash detector, Bruker, Germany). EDX spectra were quantified with the FEI software package "TEM imaging and analysis" (TIA). Using TIA, element concentrations were calculated on the basis of a refined Kramers' law model that includes corrections for detector absorption and background subtraction. Standardless quantification, i. e. by means of theoretical sensitivity factors, without thickness correction was applied. EDX spectra were taken in the STEM mode with a probe diameter of 0.5 nm. Using a focused electron probe, EDXS area scans were performed to obtain average compositions of larger sample regions. The EDX spectra were acquired by continuously scanning the electron probe in the pre-defined region.

Dynamic light scattering (DLS). DLS was used to determine the hydrodynamic diameter of the as-prepared nanocarriers in aqueous suspension. Studies were conducted at room temperature in polystyrene cuvettes applying a Nanosizer ZS (Malvern Instruments, United Kingdom).

Zeta potential measurements. Zeta potential measurements were performed using an automatic MPT-2 titrator attached to the mentioned Nanosizer ZS (Malvern Instruments,

United Kingdom). For measurements, 1 mL of the aqueous suspension containing 4.3 mg/mL of the nanocarriers were diluted in 10 mL of demineralized water and titrated with 0.1 M HCl, 0.1 M NaOH and 0.01 M NaOH.

X-ray powder diffraction (XRD). X-ray powder diffraction (XRD) was performed with a Stoe STADI-MP diffractometer (Stoe, Germany) operating with Ge-monochromatized Cu-Kα-radiation (λ = 1.54178 Å) and Debye-Scherrer geometry. The dried nanocarriers were fixed between Scotch tape and acetate paper and measured between -69° and +69° of two-theta.

Fourier-transformed infrared spectroscopy (FT-IR). Fourier-transformed infrared spectroscopy (FT-IR) was performed on a Bruker Vertex 70 FT-IR spectrometer (Bruker, Germany). All nanocarrier samples and references were pestled and diluted with KBr (3 mg of sample per 300 mg of KBr) and pressed to pellets.

Elemental analysis (C/H/N/S analysis). Elemental analysis (C/H/N/S analysis) was performed via thermal combustion with an Elementar Vario Microcube device (Elementar, Germany) at a temperature of about 1100 °C.

Optical spectroscopy (UV-Vis spectroscopy). UV-Vis spectroscopy was used to quantify the amount of ITC and UMP in the respective nanocarriers according to the Kubelka-Munk formalism. The respective concentrations were quantified in comparison to reference solutions with known concentrations by applying a calibration curve. UV-VIS spectra were recorded with an UV2700 from Shimadzu (Japan). Nanoparticle suspensions were measured in polystyrene cuvettes in an integrating sphere in diffuse transmission geometry against the corresponding pure solvent as a reference. For the preparation of the reference suspensions/solutions, see Figure S2 and the related discussion.

Fluorescence spectroscopy. A Horiba Jobin Yvon Spex Fluorolog 3.2 spectrometer equipped with a 450 W Xenon lamp, an integrating sphere (Ulbricht sphere), double grating excitation/emission monochromators, and a photomultiplier detector was used to obtain excitation and emission spectra. Nanoparticle suspensions were measured in polystyrene cuvettes located inside of the integrating sphere.

2. Synthesis of Core@Shell Nanocarriers

General aspects

Irinotecan (98 %, ABCR, Germany), dimethyl sulfoxide (≥ 99.5 %, Sigma-Aldrich, Germany), (±)-α-tocopherol phosphate disodium salt (≥ 97%, Sigma-Aldrich), ammonium acetate (97 %, VWR, Germany), zirconyl chloride octahydrate (> 99 %, Sigma-Aldrich), uridine-5´-monophosphate disodium salt (99 %, Thermo Fisher Scientific, Germany), 5-fluoro-2´-deoxyuridine 5´-monophosphate sodium salt (~85 %, Sigma-Aldrich), trisodium citrate (99 %, Carl-Roth, Germany), fluorescence red (Kremer Pigmente, Germany) and DY™-647P1-aadUTP (Dyomics, Germany) were used as purchased.

ITC@ZrO(TocP)/ZrO(FdUMP) and ITC@ZrO(TocP)/ZrO(UMP) nanocarrier. First, the ITC/TocP core was established via a solvent antisolvent approach by dissolving 2.3 mg (3.92 mmol, 1.00 eq) ITC in 0.15 mL DMSO as "solvent" and 3.9 mg (7.03 mmol, 1.79 eq) TocP in 12.0 mL demineralized water and subsequent addition of 30.0 mg (390 mmol, 99.4 eq) ammonium acetate as "antisolvent". The quick addition of the solvent to the antisolvent under ultrasound (Badelin Sonopuls HD 2070, 20 kHz, 70 W; 10 s, amplitude 100 %) in an ice bath led to a whitish, transparent, colloidally stable suspension. Subsequently, 9.0 mL of a solution of $ZrOCl_2\times 8\ H_2O$ (6.75 mg, 21.0 mmol, 5.35 eq) was added over a period of 2 min to the as prepared suspension and stirred for 10 min. After a step of centrifugation (25,000 rpm, 15 min), the precipitate was resuspended in 6 mL of an ammonium acetate solution (30 mg, 390 mmol, 99.4 eq) by ultrasonic irradiation, with an amplitude of 80% for 30 sec. During another ultrasonic treatment (30 s, amplitude 50%), 2.1 mL of a solution of Na_2(FdUMP) (1.0 mg, 3.07 mmol, 0.78 eq) for ITC@ZrO(TocP)/ZrO(FdUMP) nanocarriers or 2.1 mL of a solution of Na_2(UMP) (1.0 mg, 3.07 mmol, 0.78 eq) for ITC@ZrO(TocP)/ZrO(UMP) nanocarriers was injected after 10 s. After centrifugation (25,000 rpm, 15 min) and washing with demineralized water, the precipitate was dried or resuspended in a trisodium citrate solution (1.0 mg/mL).

ZrO(FdUMP) and ZrO(UMP) nanocarriers. 100 µL of an aqueous solution of $ZrOCl_2\times 8\ H_2O$ (5.80 mg, 18.0 mmol, 1.00 eq) was injected into 10 mL of an aqueous solution of Na_2(FdUMP) (7.36 mg, 22.7 mmol, 1.17 eq) for ZrO(FdUMP) nanocarriers or into 10 mL of an aqueous solution of Na_2(UMP) (7.36 mg, 22.7 mmol, 1.17 eq) for ZrO(UMP) nanocarriers. After 2 min of intense stirring, the ZrO(FdUMP)/ZrO(UMP) nanocarriers were separated by centrifugation (25,000 rpm, 15 min) and purified by redispersion/centrifugation

in/from H_2O. After resuspension the ZrO(FdUMP)/ZrO(UMP) nanocarriers were obtained as colloidally stable, colorless suspension.

Fluorescence Labeling. For biological studies on efficacy and cellular uptake, the nanocarriers were fluorescently labeled by the addition of small amounts of the fluorescent dyes FR or DUT647. For core labeling of ITC@ZrO(TocP)/ZrO(UMP) and ITC@ZrO(TocP)/ZrO(FdUMP), a solution of 75.0 µg FR (69.5×10^{-6} mmol, 3.86×10^{-6} eq) in 0.15 mL DMSO was used instead of pure DMSO as solvent. For shell labeling of ITC@ZrO(TocP)/ZrO(UMP) and ITC@ZrO(TocP)/ZrO(FdUMP) 12.2 µg DUT647 (10.0×10^{-6} mmol, 0.55×10^{-6} eq) was added to the solution of Na_2(UMP) or Na_2(FdUMP) solution. ITC@ZrO(TocP)/ZrO(UMP) and ITC@ZrO(TocP)/ZrO(FdUMP) nanocarriers were labeled by the addition of 48.8 µg DUT647 (40.0×10^{-6} mmol, 2.20×10^{-6} eq) to the Na_2(UMP) or Na_2(FdUMP) solution. The resulting suspensions exhibit magenta (FR) or blue (DUT647) color which are characteristic for the respective dye.

3. Material Characterization of Core@Shell Nanocarriers

The stability of ITC@ZrO(TocP)/ZrO(UMP) nanocarriers was tested over two weeks (Figure S1). If the as-prepared nanocarriers were stored in a fridge at 4 °C without stirring, the aqueous suspensions do not show any sedimentation (Figure S1a). According to DLS and SEM, particle size and size distribution of the ITC@ZrO(TocP)/ZrO(UMP) nanocarriers are similar within the significance of the experiments. Thus, DLS resulted in a mean size of 55.8±13.0 nm (as-prepared; *see main paper: Figure 4b*) and 55.5±11.8 nm (after two weeks; Figure S1b). A statistical evaluation of ≥ 100 on SEM images resulted in 41.5±13.9 nm (as-prepared; *see main paper: Figure 4a,c*) and 41.8±10.3 nm (after two weeks; Figure S1c).

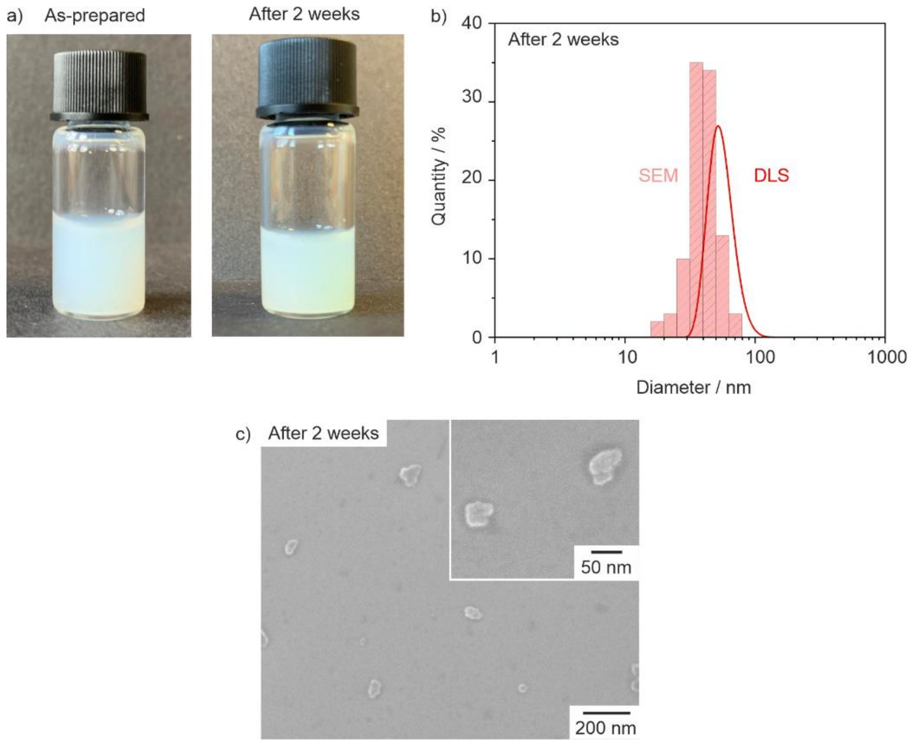

Figure S1. Stability of ITC@ZrO(TocP)/ZrO(UMP) nanocarriers after two weeks (aqueous suspension, 4 °C, non-stirred): a) Photos of aqueous suspensions after synthesis and after two weeks, b) size distribution according to DLS (in water) and SEM (statistical evaluation of ≥ 100 on SEM images) after two weeks; c) STEM image after two weeks (for DLS and SEM of the as-prepared nanocarriers *see main paper: Figure 4a-c*).

In addition to the colloidal stability and storage stability, the release profile of the ITC@ZrO(TocP)/ZrO(UMP) nanocarriers was monitored via dialysis. To this concern, the ZrO(UMP) nanocarriers (representing only the shell of the nanocarriers) and the core@shell

ITC@ZrO(TocP)/ZrO(UMP) nanocarriers were examined separately over a period of 75 hours at 37 °C.

Release profile of ZrO(UMP) nanocarriers. To monitor the release of UMP from the ZrO(UMP) nanocarriers (Figure S2a), 1.5 mL of a ZrO(UMP) suspension (3 mg/mL with 2.26 mg/mL UMP) were added to a dialysis tube (Scienova, ZelluTrans T3, 25 mm × 5 m, cut-off molecular weight 12-14 kDa) and completely immersed in the respective medium (100 mL, H_2O, pH = 6.5) and stirred at 200 rpm. At fixed time intervals, 3 mL of the solution outside the dialysis tube were separated, and the UMP content was measured by UV-Vis spectroscopy using the absorbance at λ = 260 nm. Finally, the solution was returned to the medium.

Release profile of ITC@ZrO(TocP)/ZrO(UMP) nanocarriers. To monitor the release of ITC and UMP from the ITC@ZrO(TocP)/ZrO(UMP) nanocarriers (Figure S2b), 1.5 mL of an ITC@ZrO(TocP)/ZrO(UMP) suspension (3 mg/mL with 0.66 mg/mL ITC and 0.30 mg/mL UMP) were added to a dialysis tube (Scienova, ZelluTrans T3, 25 mm × 5 m, cut-off molecular weight 12-14 kDa) and completely immersed in the respective medium (100 mL, H_2O, pH = 6.5 and pH = 4) and stirred at 200 rpm. At fixed time intervals, 3 mL of the solution outside the dialysis tube were separated and the UMP and ITC content was measured using UV-Vis spectroscopy. Here, the absorbance at λ = 360 nm (ITC) and at λ = 260 nm (UMP, TocP, ITC) was used, with the superposition of the signal at λ = 260 nm was (30 % of the signal related to UMP, 70 % of the signal related to TocP and ITC, *see Figure S3*). Finally, the solution was returned to the medium.

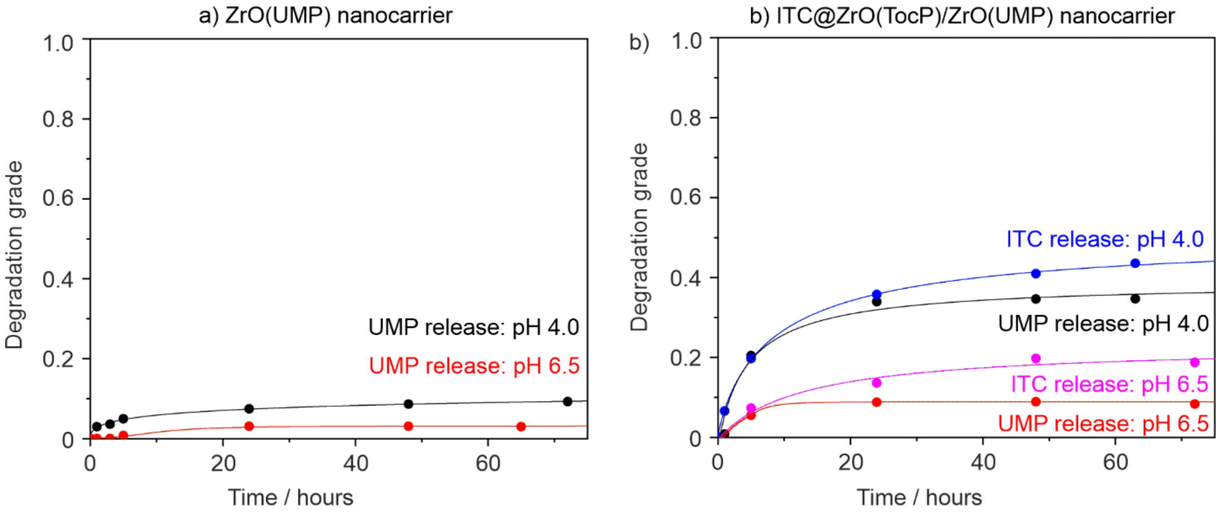

Figure S2. Stability of nanocarriers at different pH values over 75 hours at 37 °C: a) ZrO(UMP) nanocarriers at pH 6.5 with release of UMP; b) ITC@ZrO(TocP)/ZrO(UMP) nanocarriers at pH 6.5 and at pH 4.0 with release of ITC and UMP.

In sum, the nanocarriers show a faster release of the drugs in water over the first 20 hours (Figure S2). Thereafter, no or only a very slow release occurred. This finding can be rationalized based on a higher reactivity of the nanocarrier surface. In total, ZrO(UMP) nanocarriers show a release of 3 % UMP at pH = 6.5 and a release of 9 % at pH = 4.0 after 75 hours (Figure S2a). ITC@ZrO(TocP)/ZrO(UMP) nanocarriers show a release of 20 % ITC and 9 % UMP at pH = 6.5 of 43 % ITC and 38 % UMP at pH = 4.0 after 75 hours (Figure S2b). Even after 75 hours at pH 6.5, thus, the ITC@ZrO(TocP)/ZrO(UMP) nanocarriers contain 80 % of their initial ITC load and more than 90 % of their initial UMP load. The drug release is accelerated at lower pH, which reflects the dissolution of the nanocarriers in perinuclear organelles like late endosomes or lysosomes (*see main paper: Figures 5,6*).

X-ray powder diffraction (XRD) indicates the as-prepared ITC@ZrO(TocP)/ZrO(UMP) core@shell nanocarriers as amorphous (Figure S3a). Fourier-transform infrared (FT-IR) spectra indicate the presence of TocP, UMP and ITC qualitatively (Figure S3b). Thus, intense P=O vibrations (1100, 990 cm^{-1}) originate from TocP and UMP. C=O vibrations (1680 cm^{-1}) stem from UMP. Furthermore, the presence of ITC is indicated by pyridine-related vibrations (ν(C=N), ν(C=C): 1620-1600 cm^{-1}).

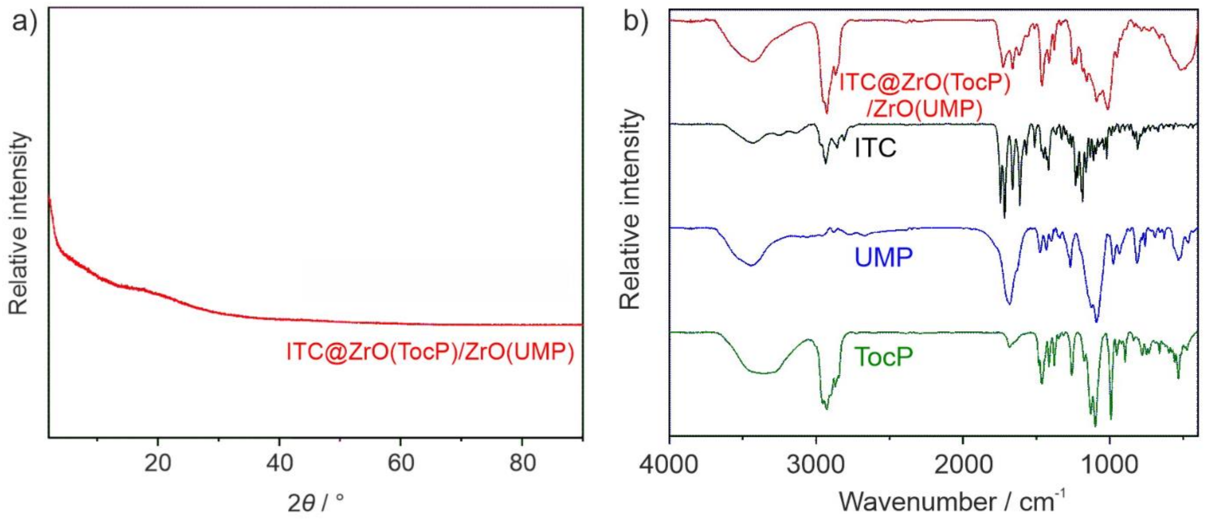

Figure S3. Chemical composition of ITC@ZrO(TocP)/ZrO(UMP) core@shell nanocarriers: a) X-ray powder diffraction of as-prepared nanocarriers, b) FT-IR spectrum of as-prepared nanocarriers with pure ITC, UMP, and TocP as references.

Total organic combustion with thermogravimetry (TG) shows total organic combustion of 68 wt-% up to 800 °C originating from the decomposition of ITC, TocP and UMP (Figure S4a).

Based on the amount and ratio of the starting materials (i.e., 3.92 mmol ITC, 7.03 mmol Na_2(TocP), 3.07 mmol Na_2(UMP)), a total organic content of 71% can be calculated, which is in agreement with the experimental data. In regard of the total organic combustion, it needs to be noticed that the combustion can be incomplete in the presence of high phosphate loads. In fact, phosphate coatings are well-known as flame retardants as they hamper the contact to oxygen. In this regard, the thermal residue indeed showed a greyish color, which indicates certain remains of amorphous carbon. This is also the background of a slightly lower carbon content observed by elemental analysis (*see main paper*).

The solid residue of the TG analysis was identified by X-ray powder diffraction (XRD) analysis to be ZrO_2 and ZrP_2O_7 (Figure S4b). As the Zr : P ratio in ZrO(TocP) as well as in ZrO(UMP) is 1 : 1, the Zr : P ratio in the thermal residue can be expected to be 1 : 1, too. Based on the intensity of the Bragg reflections, the ratio of ZrO_2 : ZrP_2O_7 can be estimated to 1 : 1, resulting in a Zr : P ratio of 1 : 1 (Figure S4b). However, it must be taken into account that certain sublimation of P_4O_{10} is observed at 800 °C.

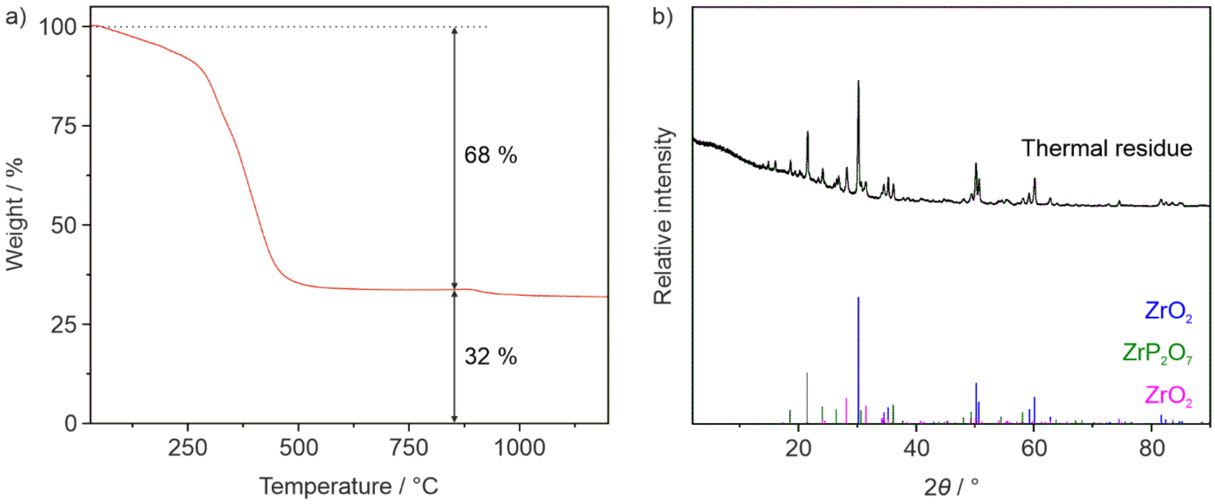

Figure S4. Chemical composition of ITC@ZrO(TocP)/ZrO(UMP) nanocarriers: a) TG, b) XRD of TG residue (ZrO_2: ICDD-No. 01-072-7115, ZrO_2: ICDD-No. 01-070-2491, ZrP_2O_7: ICDD-No. 00-049-1079 as references).

The drug load of the ITC@ZrO(TocP)/ZrO(UMP) nanocarriers was examined by photometry. To this concern, UV-VIS spectra were recorded (Figure S5a). Herein, the characteristic absorbance of ITC (360 nm) and UMP (260 nm) were used for comparison with certain reference samples following the Lambert-Beer law. The reference samples with ITC/TocP suspended in water and UMP dissolved in water show a good linear correlation of optical absorption and concentration (Figure S5b). The respective concentrations were

quantified in comparison to reference suspensions/solutions with known concentrations by applying calibration curves. For ITC/TocP, suspensions with concentrations 15.0, 5.0, 1.67, 0.56, 0.19 µg/mL and an absorbance (at 360 nm, Figure S5a) of 0.26, 0.085, 0.032, 0.012, 0.006, resulting in a calibration curve gradient of 0.01733 $\mu g^{-1} mL^{-1}$ were used (Figure S5b). For UMP, solution with concentrations of 16.70, 5.60, 1.85, 0.60, 0.21 µg/mL and an absorbance (at 260 nm, Figure S5a) of 0.353, 0.118, 0.042, 0.020, 0.005, resulting in a calibration curve gradient of 0.0212 $\mu g^{-1} mL^{-1}$ (Figure S5b). ITC@ZrO(TocP)/ZrO(FdUMP) and ITC@ZrO(TocP)/ZrO(UMP) nanocarriers were measured with a dilution factor of 280× (measuring point: absorbance 0.18 at 360 nm) for ITC quantification. The references, including ZrO(FdUMP) nanocarriers, ZrO(UMP) nanocarriers, FdUMP solution as well as the supernatants of the ITC@ZrO(TocP)/ZrO(FdUMP) and ITC@ZrO(TocP)/ZrO(UMP) nanocarriers were measured with a dilution factor of 200× for FdUMP or UMP quantification (measuring point: absorbance 0.14 at 260 nm). With the resulting calibration, photometry results in 2.9 mg/mL of ITC and 1.3 mg/mL of UMP at a particle concentration of 13 mg/mL, which corresponds to drug loads of 22 wt-% ITC and 10 wt-% UMP.

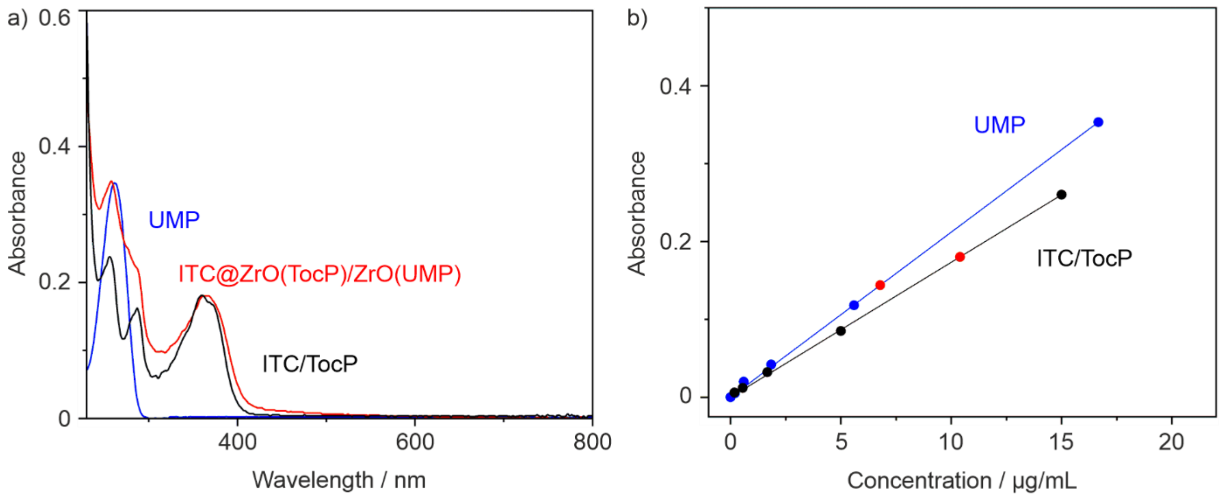

Figure S5. Photometric analysis of ITC@ZrO(TocP)/ZrO(UMP) nanocarriers: a) UV-VIS spectra with an ITC/TocP solution in water and UMP solution in water as references, b) calibration curves to extract the ITC and UMP concentration in the nanocarriers (red dots).

4. *In vitro* Characterization

Cell culture

All cell lines were from the American Type Culture Collection (ATCC, Manassas, VA, USA). Human colon cancer cell line HCT116 were maintained in McCoy's media (Gibco) supplemented with 10 % Fetal Bovine Serum (FBS) and 10 µg/mL Penicillin/Streptomycin (Sigma Aldrich, P4333). Human immortalized retinal pigment epithelial cell line RPE-1 were grown in Dulbecco's Modified Eagle Medium F12 (DMEM/F12, Gibco) supplemented with 10 % FBS and 0.01 mg/mL Hygromycin B. Human rectal tumor cell lines SW1463, SW837 were grown in Leibovitz's media supplemented with L-Glutamine, 10 % FBS, and 10 µg mg/mL Penicillin/Streptomycin. The cell lines were passaged and grown at 37 °C in a humidified atmosphere with 5 % CO_2.

Patient-derived organoids (PDO) culture

Fresh tissue samples used in this study were provided by the University Medical Center Göttingen (UMG), Germany. The study was approved by the Ethical Committee of the University Medical Center Göttingen (UMG Antragsnr. 25/3/17). Rectal cancer patient-derived organoids were established and cultured according to Sato et al. 2011 and De Oliveira *et al*, 2021. In short, tumor organoids were cultured in advanced DMEM/F12 media supplemented with 1×Glutamax, 10 mM Hepes, 1×10 µg/mL of penicillin/streptomycin (all from Gibco), 10 mM nicotinamide (Sigma), 1×B27 (Gibco), 500 nM A83-01 (Tocris), 10 µM SB202190 (MedChem Express, Monmouth Junction, NJ, USA), 1.25 mM N-acetylcysteine (Sigma), 20 % R-spondin conditioned media (CM) (in house made), 10 Noggin CM (in house made), 1×N_2 supplement (Gibco), 100 µg/mL Primocin (InvivoGen, Toulouse, France) and 50 ng/mL human epidermal growth factor (EGF) (Gibco). 10 µM Y-27632 (Adooq Biosciences, Irvine, CA, USA) was added to the medium after organoid extraction.

For experiments, rectal cancer PDOs were dissociated into single cells and 10,000 cells were seeded in 10 % matrigel in the PDO tumor media (v/v) in a 96-well plate. The plate was incubated for 3 days to allow PDOs to grow to reach a uniform size. Free drugs and NPs were diluted in PDO tumor media and then added to the wells. After 5 days of treatment, PDOs were analyzed by incucyte cell live imaging and CellTiterGlo assay.

Cellular uptake assay

30,000 cells were seeded in a 24-well plates containing poly-lysine coated coverslips. After allowing proper cell attachment and growth for 2 days, cells were treated with REF-NC either

for 4 or 24 h. Cells were washed 3-times with (phosphate buffer solution) PBS to remove residual REF-NC that were not internalized by cells. Consequently, cells were fixed in 4 % paraformaldehyde (PFA) and stained then with Hoechst33342 (λ_{ex} = 405 nm, λ_{em} = 415-479 nm) (Thermo Fischer Scientific, 62249), 800 nM Cell Mask Green (λ_{ex} = 522 nm, λ_{em} = 533-637 nm) (C37608, Thermo Fischer Scientific) diluted in PBS. The cells were imaged with a Leica SP8 confocal microscope (Plan Neofluor 63×/oil NA 1.4 objective with 1 Airy Unit). Staining and microscopy conditions were kept identical for comparisons. The number of puncta/cell quantifications were performed using available pipelines with some modifications in CellProfiler (Broad Institute of MIT and Harvard). In brief, the cells were segmented with cell mask green staining as the primary object and REF-NC fluorescence signal (DUT647, λ_{ex} = 651 nm, λ_{em} = 673 nm) as the secondary object. The number of REF-NC puncta per cell was obtained by number of secondary objects (REF-NC puncta) overlapping the cell mask green primary object.

LysoTracker staining and immunostaining

30,000 cells were seeded in a 24-well plates containing poly-lysine coated coverslips. After allowing proper attachment and cell growth for 2 days, cells were treated with reference REF-NC either for 4 or 24 h. 500 nM of LysoTracker Red DND-99 red (L7528, Thermo Fischer Scientific, λ_{ex} = 577 nm, λ_{em} = 588-637 nm) was added to live cells. After 20 min incubation in the cell culture incubator, cells were washed 3 times with PBS to remove residual REF-NC and LysoTracker dye that were not internalized by cells. Consequently, cells were fixed in 4 % PFA and stained then with Hoechst33342.

For immunostaining with antibodies, cells were were fixed and permeabilized with 4 % PFA and 0.2 % Triton-X-100. The cells were then blocked in 3 % bovine serum albumin diluted in PBS, followed by 90 min incubation with primary antibodies. The primary antibodies used in this study are rabbit anti-Rab5 (1 : 100, PA5-29022, Invitrogen), rabbit anti-Rab7A (1 : 100, HPA006964Y, Sigma), rabbit anti-Rab11 (1 : 100, 700184, Invitrogen), mouse anti-LAMP1_Alexa Fluor 488 (1 : 250, H4A3, Invitrogen). After washing three times, cells were incubated with secondary antibodies (Molecular Probes) conjugated to Alexa-Fluor-488 or 546 and Hoechst33342 to label nuclei (Figure S6). Staining and microscopy conditions were kept identical for comparisons. The cells were imaged with a Leica SP8 confocal microscope (plan Neofluor 63×/oil NA 1.4 objective with 1 Airy Unit).

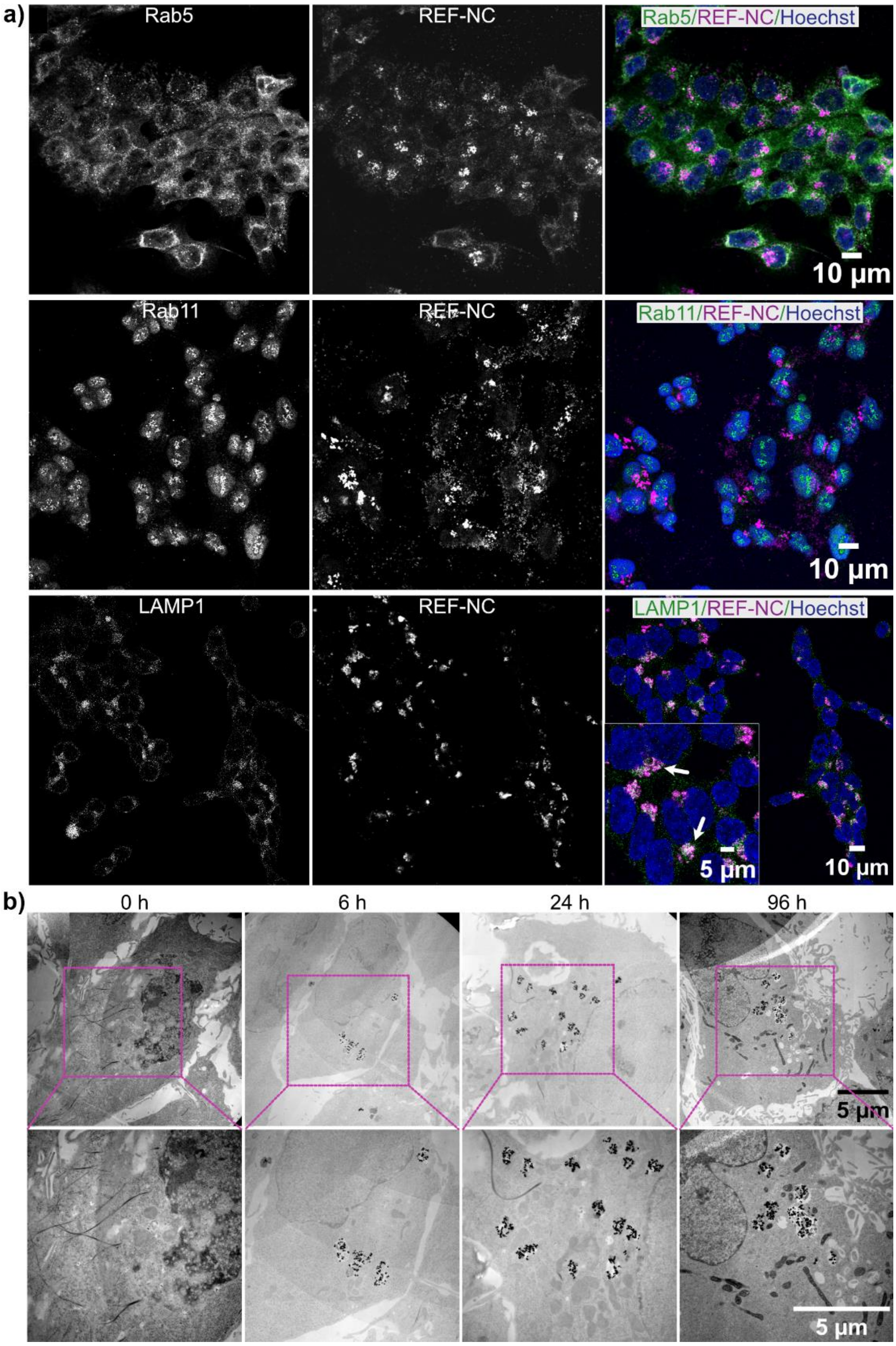

Figure S6. Cellular uptake and intracellular trafficking of nanocarriers: a) Confocal microscopy images of HCT116 treated with 230 µg/mL of REF-NC for 24 h, and cells immunostained with antibodies against either Rab5 (upper panel), Rab11 (middle panel), or LAMP1 (lower panel); nuclei labeled with Hoechst 33342. The arrows in the LAMP1 staining indicate colocalization (white) between LAMP1 and REF-NC. b) Representative transmission electron microscopy images of HCT116 cells untreated (0 h) or treated with 230 µg/mL of REF-NC for 6, 24, and 96 h shown at two different magnifications.

Live cell-imaging to study nanocarrier degradation

5000 cells were seeded in an 8-well slide IBIDI chamber, after allowing proper attachment and growth for 2 days, cells were pretreated with 57.5 µg/mL of reference REF-NC overnight. Non-internalized nanoparticles were removed by washing the cells three times with PBS. Consequently, the intracellular REF-NC degradation over time was imaged by using a Nikon spinning disk confocal microscope with 40 X air objective for 48 h. The number of puncta per image and mean fluorescence intensity of REF-NC over time was calculating by analyzing the time-lapse movies of cells (brightfield) with REF-NC fluorescent signal in three dimensions (3D) using IMARIS. To identify REF-NC puncta and their mean fluorescence intensity, the fluorescent signal from DUT647 (λ_{ex} = 651, λ_{em} = 673 nm) was segmented using the "spots" function of Imaris and then data were exported into excel.

Live cell-imaging to study uptake of nanocarriers by PDOs

For live cell imaging experiments, rectal cancer PDOs were dissociated into single cells and 10,000 cells were seeded in 10 % matrigelin the PDO tumor media (v/v) in an 8-well IBIDI slide chamber. The slide chamber was incubated for 4 days to allow PDOs to grow to reach a uniform size. REF-NPs were diluted in PDO tumor media and then added to the wells. After 24 h of treatment, PDOs were imaged in an inverted Nikon spinning disk confocal microscope using a 40× air objective.

CellTiter-Glo® luminescent viability assay

Cell viability was measured by performing a CellTiter-Glo assay (Promega, G8461). Cells were seeded in a 96-well plate. After drug treatment for the indicated duration, 80 μL of the cell titer glow reagent (Promega) diluted to 1 : 5 with PBS were added to each well. The plate was incubated on a shaker for 2 min at room temperature to allow cell lysis and then incubated without shaking for 10 min at RT to allow luminescence signal stabilization. The signal was measured using a multimode plate reader CLARIOstar (BMG LABTECH, Germany).

Incucyte imaging

The real-time proliferation of the cells was detected by the Incucyte® SX5 Live Cell Analysis System (Sartorious) by imaging each well at 2 h intervals for 72 hours with a 10× objective. The images were analyzed using the inbuilt incucyte software "AI cell Health" which allows label-free live-cell segmentation. The proliferation kinetics were obtained by calculating live cell count at each time point normalized to the control untreated condition. For PDOs, each

well was imaged with a 4× objective at 2 h intervals for 5 days. The images then analyzed by using the incucyte "organoid" analysis function which measures PDO object area per image and PDO object count (Figure S7).

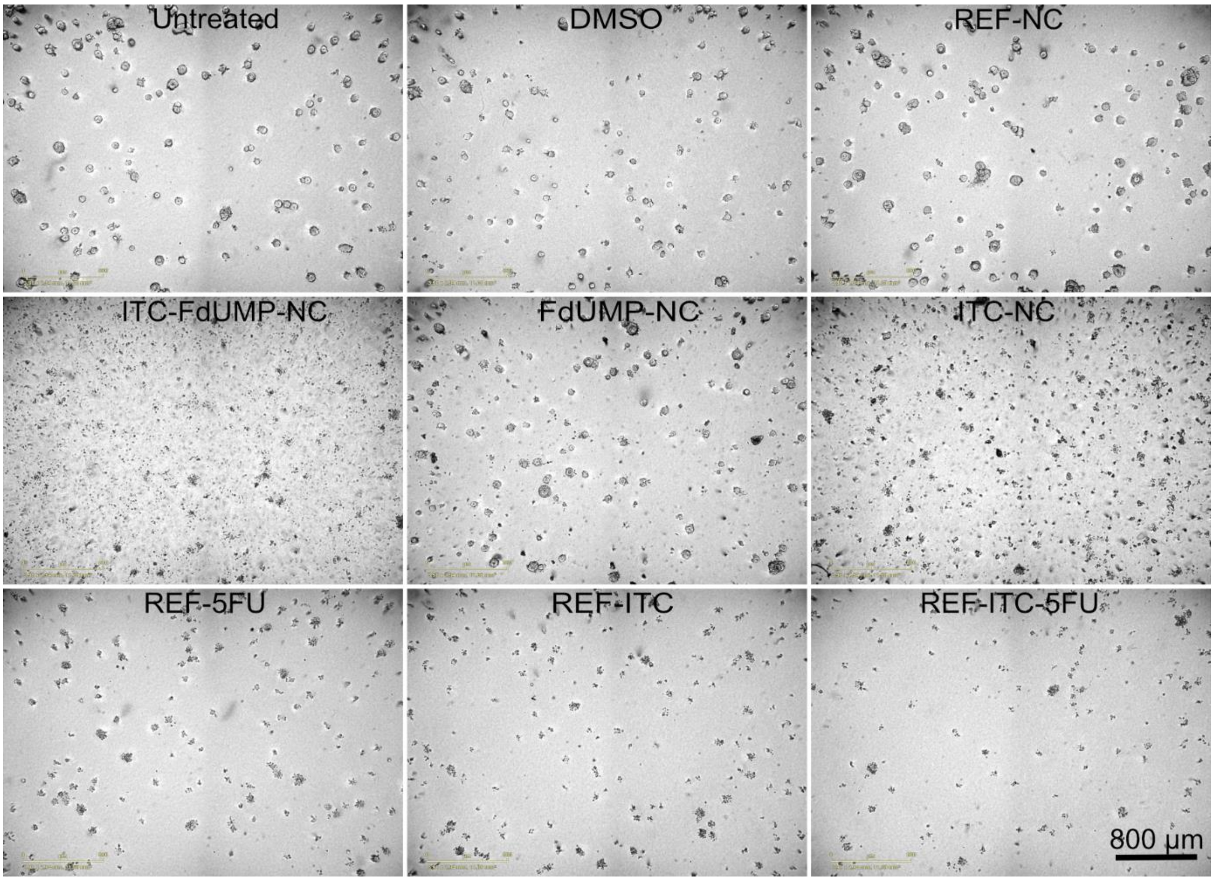

Figure S7. Potency of FOLFIRI-type ITC-FdUMP-NC and the free drugs in rectal cancer patient-derived organoids (PDO): Representative incucyte images of an organoid developed from rectal cancer of a patient, grown in a media with 10 % Matrigel(v/v) and treated for 5 days either with different controls (untreated, DMSO, REF-NC), or the single free drugs ITC (80 µM), 5FU (40 µM), or with a combination of ITC and FdUMP either in core@shell NC or of free drugs.

Electron microscopy

400,000 HCT116 cells were seeded in a 35 mm culture dish. After 2 days, cells treated with 230 µg/mL of reference REF-NC for 4 or 24 h and then fixed in culture dishes with 4 % formaldehyde and 2.5 % glutaraldehyde in 0.1 M phosphate buffer pH 7.4 followed by postfixation with 1 % OsO_4 (Science Services, Germany) in 0.1 M phosphate buffer at 4°C and embedded in Epon (Serva, Germany) after dehydration with ethanol and en-bloc staining with 1.5 % uranyl acetate (Merck)/1.5 % tungstophosphoric acid (Merck, Germany) in 70 % ethanol.

Ultrathin sections of cultured cells were cut parallel to the substrate using an UC7 Ultramicrotome (Leica, Germany) and stained with UranylLess® (Science Services, Germany). Sections were analyzed with a LEO EM912 Omega (Zeiss, Germany) and digital micrographs were obtained with an on-axis 2048 × 2048 CCD camera (TRS).

Statistics

Data were analyzed using GraphPad Prism 10 built-in tests. All data are presented as mean±S.D. Details about the significance test, the number of replicates, and the *P* values are reported in the respective Figure legends.
